# Genome-wide association studies for fatty acid metabolic traits in five divergent pig populations

**DOI:** 10.1038/srep24718

**Published:** 2016-04-21

**Authors:** Wanchang Zhang, Bin Yang, Junjie Zhang, Leilei Cui, Junwu Ma, Congying Chen, Huashui Ai, Shijun Xiao, Jun Ren, Lusheng Huang

**Affiliations:** 1 State Key Laboratory of Pig Genetic Improvement and Production Technology, Jiangxi Agricultural University, Nanchang, 330045, P.R. China

## Abstract

Fatty acid composition profiles are important indicators of meat quality and tasting flavor. Metabolic indices of fatty acids are more authentic to reflect meat nutrition and public acceptance. To investigate the genetic mechanism of fatty acid metabolic indices in pork, we conducted genome-wide association studies (GWAS) for 33 fatty acid metabolic traits in five pig populations. We identified a total of 865 single nucleotide polymorphisms (SNPs), corresponding to 11 genome-wide significant loci on nine chromosomes and 12 suggestive loci on nine chromosomes. Our findings not only confirmed seven previously reported QTL with stronger association strength, but also revealed four novel population-specific loci, showing that investigations on intermediate phenotypes like the metabolic traits of fatty acids can increase the statistical power of GWAS for end-point phenotypes. We proposed a list of candidate genes at the identified loci, including three novel genes (*FADS2*, *SREBF1* and *PLA2G7*). Further, we constructed the functional networks involving these candidate genes and deduced the potential fatty acid metabolic pathway. These findings advance our understanding of the genetic basis of fatty acid composition in pigs. The results from European hybrid commercial pigs can be immediately transited into breeding practice for beneficial fatty acid composition.

Fatty acids are basic energy sources and indispensable components for cellular regulation and metabolism in animals. Fatty acids also play critical roles in meat nutrition, sensory, tenderness and taste flavor^1^. For instance, polyunsaturated fatty acids (PUFA), such as linoleic acid (C18:2n6) and γ-linolenic acid (C18:3n6), are beneficial for human health by reducing low density lipoprotein cholesterol in blood^2^. Hence, a high ratio of polyunsaturated to monounsaturated fatty acids (PUFA/MUFA) and that of *omega*-3 to *omega*-6 fatty acid (n-3/n-6) are more preferable in terms of meat nutriology^3^. Although fatty acid composition is an important trait, it is usually not considered in pig breeding schemes as phenotypic measurement of this trait is time-consuming and costive. It is thus worthwhile to understand the genetic mechanisms underlying fatty acid composition in pork, which would allow us to establish novel molecular breeding tools for optimizing fatty acid composition in pork.

Recently, genome-wide association studies (GWAS) have made a substantial progress in identifying genetic factors underlying or associated with complex traits. In pigs, GWAS have detected a large number of genetic loci for a variety of phenotypic traits in divergent populations, including a list of significant loci for fatty acid composition in pork^4^,^5^,^6^,^7^. Many loci have pleiotropic effects on multiple fatty acid contents that show close phenotypic and genetic correlations with each other^4^,^5^,^6^,^7^. For these correlated traits, multivariate association models have been developed to detect and fine map the pleiotropic loci^8^.

Metabolic phenotypes are important proxies of many biological processes and pathways, including fatty acid composition. In human, GWAS have been conducted on phenotypic ratios in metabolomics^9^. To date, many loci are known to be associated with metabolic phenotypic ratios, such as serum metabolic ratios in humans^10^. It has been shown that the detection power can be raised several orders of magnitudes when metabolic ratios are used as quantitative traits in GWAS^10^.

The aim of this study was to detect genomic loci for fatty acid metabolic traits in five pig populations that remain unexplored in our previous studies^5^,^6^,^7^. We first tested phenotypic correlations between fatty acid metabolic traits. By applying GWAS, we then identified several novel pleiotropic loci for the tested traits and highlighted a list of candidate genes for the identified loci. We further built a potential network and pathway in which these candidate genes were involved. Our findings provide insight into the genetic mechanism of fatty acid composition in pork.

## Materials and Methods

### Ethics statement

All the experiments that involved animals were carried out in accordance with the approved guidelines by the Ministry of Agriculture of China. Approval was obtained from the ethics committee of Jiangxi Agricultural University before this study.

### Animals and phenotypes

Five pig populations were used in this study, including one White Duroc × Erhualian F_2_ intercross (hereafter referred to as F_2_), one Duroc × (Landrace × Yorkshire) hybrid (hereafter referred to as DLY) population, and three Chinese pig populations (Sutai, Erhualian and Laiwu). The pedigree, management and phenotype recording information about the five populations has been described in our previous publications^5^,^6^,^7^,^11^,^12^. Briefly, two White Duroc founder boars and 17 Chinese Erhualian founder sows were mated to produce F_1_ animals, from which nine F_1_ boars and 59 F_1_ sows were intercrossed to produce 976 F_2_ males and 945 F_2_ females in six batches. Fatty acid composition traits were measured on *longissimus dorsi* muscle and abdominal fat samples of 677 F_2_ pigs. Sutai is a Chinese synthetic pig line that was originally generated from a cross between Chinese Erhualian/Meishan and Duroc pigs and has experienced artificial selection for prolificacy and growth for more than 18 generations^13^. A total of 281 Sutai pigs from five sires and 60 dams were measured for fatty acid composition in *longissimus dorsi* muscle as described previously^5^. Erhualian pigs that was originally located in Wuxi, Jiangsu Province, are famous for their high litter size of greater than 16^13^. Laiwu is a Chinese indigenous pig breed that was originally distributed in Laiwu, Shangdong Province. The Laiwu pig is known for its unusually high intramuscular fat content (average 9~12%)^13^. We measured fatty acid composition on *longissimus dorsi* muscle samples of 336 Erhualian pigs from 11 sires and 55 dams and 333 Laiwu pigs from 25 sires and 115 dams that covered the majority of sire and dam lines of the two Chinese breeds. During the fattening period (from ~90 days to the slaughter ages), F_2_, Sutai, Erhualian and Laiwu pigs were raised in a pig farm in Nanchang, Jiangxi Province, and were *ad libitum* fed the same diet containing 16% crude protein, 3100 kJ digestible energy and 0.78% lysine under standard management conditions. A total of 698 DLY pigs at 180 ± 3 days were purchased from a commercial pig farm in Xiushui, Jiangxi Province. DLY boars were castrated at day 25. All DLY pigs were raised under a consistent diet containing 16% crude protein, 2132 digestible energy and 0.85% lysine. Finally, F_2_ and Sutai pigs at 240 ± 3 days, DLY pigs at 180 ± 3 days, and Erhualian and Laiwu pigs at 300 ± 3 days were slaughtered in the same commercial abattoir. After slaughter, *longissimus dorsi* muscle samples from all individuals and abdominal fat tissues from the F_2_ pigs were collected to measure fatty acid composition as described previously^5^,^6^,^7^. In this study, we further calculated five fatty acid metabolic indices using the following equations^14^,^15^:


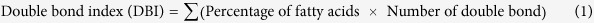



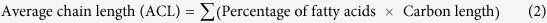



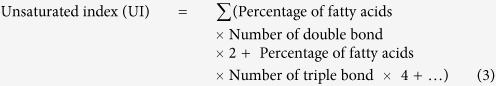



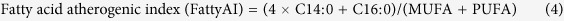



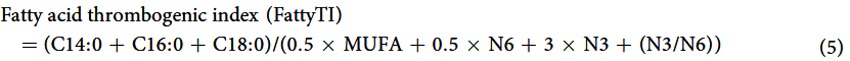


where MUFA is monounsaturated fatty acids including myristoleic acid (C14:1n5), palmitoleic acid (C16:1n7), oleic acid (C18:1n9) and eicosenoic acid (C20:1n9), and PUFA is polyunsaturated fatty acids including linoleic acid (C18:2n6), γ-linolenic acid (C18:3n6), α-linolenic acid (C18:3n3), eicosadienoic acid (C20:2n6), dihomo- γ-linolenic acid (C20:3n6), eicosatrienoic acid (C20:3n3), arachidonic acid (C20:4n6); N3 is *omega*-3 PUFA comprising C18:3n3, C20:3n3, and N6 is *omega*-6 PUFA including C18:2n6, C18:3n6, C20:2n6, C20:3n6 and C20:4n6.

We also determined the ratios of UFA to SFA (UFA/SFA), PUFA to MUFA (PUFA/MUFA), MUFA to SFA (MUFA/SFA), PUFA to SFA (PUFA/SFA) and N6 to N3 (N6/N3), where UFA refers to unsaturated fatty acids including MUFA and PUFA, and SFA is saturated fatty acids including myristic acid (C14:0), palmitic acid (C16:0), stearic acid (C18:0) and arachidic acid (C20:0). Lastly, we calculated all possible products/substrates ratios in fatty acid metabolic pathway, including C14:1n5/C14:0, C16:0/C14:0, C18:0/C16:0, C16:1n7/C16:0, C18:1n9/C16:1n7, C18:1n9/C18:0, C18:3n6/C18:2n6, C20:0/C18:0, C20:1n9/C18:1n9, C20:1n9/C20:0, C20:2n6/C18:2n6, C20:3n6/C18:2n6, C20:3n6/C18:3n6, C20:3n3/C18:3n3, C20:4n6/C18:2n6, C20:4n6/C18:3n6, C20:4n6/C20:2n6 and C20:4n6/C20:3n6 (Supplementary Table 1). Before the association analysis, 33 fatty acid metabolic traits were log_2_-transformed owing to the relationship of log (a/b) = −log (b/a), so that the association of a ratio and of its inverse would obtain identical results.

### Genotypes and quality control

Genomic DNA was extracted from ear tissue using a standard phenol-chloroform method. As reported previously^5^,^7^,^11^,^12^, animals from the DLY, Erhualian and Laiwu populations were genotyped with PorcineSNP60 BeadChips (v2), and those from the F_2_ and Sutai populations were genotyped with BeadChip (v1)^16^ on an iScan system (Illumina, USA) following the manufacture’s instruction. SNPs with call rates > 0.90 and minor allele frequencies > 0.01, samples with genotyping call rates > 0.90 were retained for further statistical analyses. To compare the current results to our previously reported loci for fatty acid contents, we re-conducted GWAS for fatty acid composition using the same above-mentioned quality control procedures across the five populations. A meta-analysis of GWAS was conducted for each fatty acid metabolic index by using a common set of qualified SNPs across the five populations.

### Statistical analyses

To investigate phenotypic correlations between the metabolic traits measured, we corrected phenotypes by treating sex and slaughter batch as fixed effects, and polygenic effects as random effects under a linear mixed model implemented in *polygenic* function in R package GenABEL^17^. The phenotypic correlation matrix was calculated by Pearson product-moment correlation coefficients using “corrplot” package in R. The *polygenic* function of GenABEL package was applied to estimate heritabilities of fatty acid metabolic traits^17^. The phenotypic variance explained by each top GWAS SNP was calculated by (V_reduce_ − V_full_)/V_reduce_, where V_reduce_ and V_full_ are residual variances of ordinary linear models with and without including SNP genotypes as predictor variables, respectively.

For GWAS, single-marker association was conducted using *polygenic* and *mmscore* function of GenABEL. A generalized linear mixed model (see equation (6)) was explored to evaluate the association between qualified SNPs and phenotypic values.


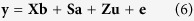


where **y** is the vector of phenotypes; **b** is the vector of fixed effects including sex and batch effects; **a** is the allelic substitution effects; **u** is the vector of random additive genetic effects following the multinormal distribution **u** ~ N (0, **G**σ_α_^2^), in which **G** is the genomic relationship matrix that was constructed based on qualified SNPs, σ_α_^2^ is the polygenetic additive variance. **X** and **Z** are the incidence matrices for **b** and **u**, respectively; **S** is the incidence vector for **a**, and **e** is a vector of residual errors with a distribution of N (0, **I**σ_e_^2^); where **I** is the identity matrix and σ_e_^2^ is the residual variance. Population stratification was accounted by adjusting the kinship covariance matrix estimated from SNP data using “ibs” function in GenABEL. Genomic inflation factors (λ) were calculated as *median* (obsPvals)/*median* (expPvals), where “obsPvals” and “expPvals” represent the observed and expected *P* values in the association analysis, respectively. Sex and batch were fitted as fixed effects in the model. Bonferroni methods were used to correct the genome-wide significant (0.05/N) and suggestive (1/N) thresholds, where N is the number of SNPs used for analyses.

A meta-analysis was conducted using a Z-score method implemented in METAL software^18^, which combined the *P*-value and allelic effect of each SNP. A total of 28,124 common SNPs across the five populations were analyzed in the meta-analysis.

### Gene function, pathway and network analyses

To highlight functionally plausible candidate genes at genome-wide significant loci, we operationally searched for annotated genes within a 1 Mb region centering each top SNP on the pig reference genome assembly (Build 10.2). The homologous segments in other mammals including mouse, cattle and human were investigated to characterize unannotated genes in target regions. LASTZ, a sequence alignment program^19^, was explored to align pairwise genomic sequences of different species. Candidate genes were determined by their functions related to fatty acid metabolism through literature mining. Enriched functional connections and networks involving the highlighted candidate genes were investigated by using GeneMANIA^20^. A potential fatty acid metabolic pathway was deduced from the known biochemical functions of the highlighted candidate genes.

## Results

### Summary of phenotype and genotype statistics

Supplementary Table 1 shows phenotypic values of 33 fatty acid metabolic traits measured in the five populations, including number of individuals, mean, standard deviation and estimated heritabilities (*h*^ 2^). Most (F_2_ longssimus muscle, 70%; F_2_ abdominal fat, 85%; Sutai, 42%; Erhualian, 82%; Laiwu, 82%; DLY, 55%) of the metabolic indices displayed moderate to high heritability estimates ranging from 0.25 to 0.65, indicating that genetic components significantly contribute to fatty acid metabolic traits. We estimated phenotypic correlations between the 33 analyzed traits (Supplementary Fig. 1). SFA, FattyAI and FattyTI were highly correlated with UFA/SFA, MUFA/SFA, DBI and UI because of the definitive calculation methods of these traits. After the same quality control process, a total of 47,953, 49,109, 56,126, 35,974 and 49,442 SNPs were qualified for 597 F_2_, 281 Sutai, 610 DLY, 336 Erhualian and 319 Laiwu pigs. The average adjacent distances between markers were 62.56, 61.08, 53.45, 83.39 and 60.67 kilo bases (kb) in the F_2_, Sutai, DLY, Erhualian and Laiwu populations, respectively. Bonferroni-corrected genome-wide significant thresholds were 1.04 × 10^−6^ (0.05/47,935), 1.01 × 10^−6^ (0.05/49,109), 8.91 × 10^−7^ (0.05/56,126), 1.39 × 10^−6^ (0.05/35,974) and 1.01 × 10^−6^ (0.05/49,442) in these populations, respectively.

### Single-population GWAS

We conducted single-marker association studies on the 33 fatty acid metabolic traits measured in the five populations. In total, we detected 11 genome-wide significant loci on nine chromosomes (Table 1) and 12 suggestive regions on nine chromosomes (Table 2). Of the 11 significant loci, four were population-specific and novel loci that were not identified in previous studies^5^,^6^,^7^, including the *Sus scrofa* chromosome (SSC) 2 locus (9.13–12.86 Mb) for C20:3n6/C18:2n6 and C20:4n6/C20:3n6 in Erhualian, the SSC12 locus (57.80–61.49 Mb) for C20:4n6/C20:3n6, PUFA/SFA and SFA in Laiwu, the SSC13 locus (28.52 Mb) for C16:1n7/C16:0 in DLY and the SSCX locus (124.40–125.87 Mb) for C20:2n6/C18:2n6 and C20:4n6/C20:2n6 in Laiwu. The other seven loci corresponded to previously identified loci for fatty acid composition^5^,^6^,^7^,^21^,^22^. These loci were distributed on SSC2, 7, 8, 12, 14, 16 and X and showed pleiotropic effects on more than one fatty acid metabolic trait (Table 1). Three of these loci were also evidenced as pleiotropic QTL for fatty acid composition in both abdominal fat and *longissimus dorsi* tissues in the F_2_ population in our previous studies^5^, including the SSC7 locus (27.45–80.45 Mb), the SSC8 locus (119.42–129.56 Mb) and the SSC16 locus (21.23–63.72 Mb) (Table 1). Moreover, we detected several loci simultaneously affecting the same metabolic index in a single population. For instance, the loci at 27.45–80.45 Mb, 134.27–134.68 Mb on SSC7 and 21.23–63.72 Mb on SSC16 affected C20:1n9/C20:0 in abdominal fat tissue in the F_2_ population, and the loci at 119.72–121.47 Mb on SSC8, 28.52 Mb on SSC13 and 120.21–123.29 Mb on SSC14 affected C16:1n7/C16:0, apparently indicating the polygenic basis of these traits.

**Figure 1:**
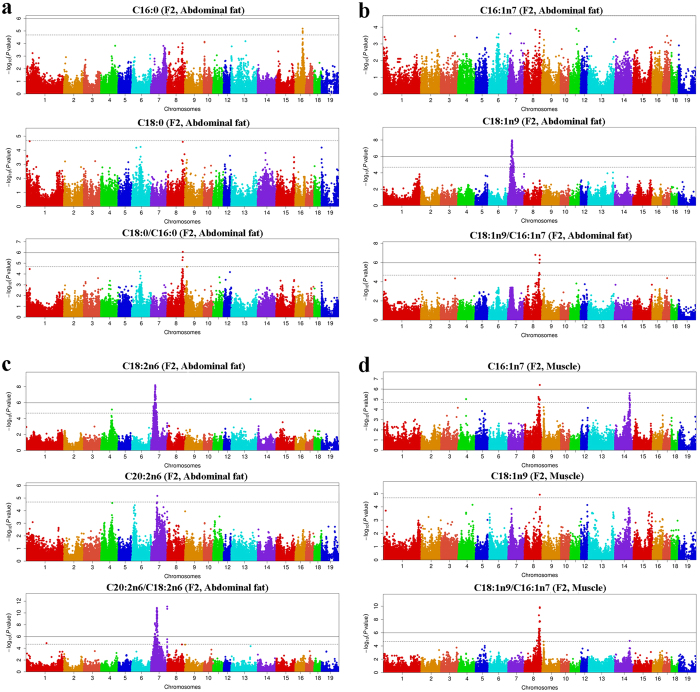
Manhattan plots of significant loci for fatty acid metabolic traits in the White Duroc × Erhualian F_2_ population. (**a**) GWAS results for C16:0, C18:0 and C18:0/C16:0 in abdominal fat; (**b**) GWAS results for C16:1n7, C18:1n9 and C18:1n9/C16:1n7 in abdominal fat; (**c**) GWAS results for C18:2n6, C20:2n6 and C20:2n6/C18:2n6 in abdominal fat; (**d**) GWAS results for C16:1n7, C18:1n9 and C18:1n9/C16:1n7 in *longissimus dorsi* muscle. Negative log_10_ *P* values of the qualified SNPs (N) are plotted against their genomic positions. SNPs on different chromosomes are denoted by different colors. The solid line represents the genome-wide threshold (0.05/N). The dash line indicates the suggestive threshold (1/N).

In the F_2_ population, a total of 441 SNPs on seven chromosomal regions were significantly associated with 25 fatty acid metabolic traits. We detected a genome-wide significant locus for abdominal fat weight in the F_2_ population^23^. Considering that fatty acid composition in abdominal fat tissues was correlated with abdominal fat weight in this population^24^, we herein treated abdominal fat weight as a fixed effect in the GWAS model and found no obviously different results (data not shown). This indicates that the loci affecting abdominal fat weight have no significant effect on fatty acid composition in abdominal fat tissues. In our previous study, we detected only one suggestive locus at 52.35 Mb on SSC16 for C16:0 content and did not find any significant loci for C18:0 content in abdominal fat tissues in the F_2_ population^5^. When we used the ratio of C18:0/C16:0 as a phenotype, we herein detected a region at 126.83–129.56 Mb on SSC8 that showed genome-wide significant (*P* < 8.91E-7) association with the metabolic trait with the top SNP (ASGA0039796) at 129.44 Mb on this chromosome (Fig. 1a). We previously identified a significant (*P* = 5.07E-08) locus around 34.80 Mb on SSC7 for C18:1n9 but not for C16:1n7 content in abdominal fat^5^. However, when GWAS was re-conducted for the ratio of C18:1n9/C16:1n7, all the significant SNPs associated with C18:1n9 on SSC7 disappeared. Instead, a significant region at 128.84–129.56 Mb on SSC8 with the top SNP (ALGA0049404, *P* = 1.81E-07) was detected for the metabolic ratio (Fig. 1b). At 134.27–134.68 Mb on SSC7, we identified a significant locus for the ratio of C20:2n6/C18:2n6 in abdominal fat tissues in the F_2_ population (Fig. 1c). This region was not associated with C18:2n6 or C20:2n6 content in the F_2_ population. A marked locus containing one significant and five suggestive SNPs around 129 Mb on SSC8 have been associated with C16:1n7 content in *longissimus dorsi* muscle in the F_2_ population^5^. The top SNP (H3GA0025376, *P* = 3.97E-07) at 126.88 Mb explained 6.24% of phenotypic variance^5^. Here we conducted GWAS for C18:1n9/C16:1n7, resulting in more associated SNPs (N = 14) in the 119–130 Mb region on SSC8 and an increased association strength with the top SNP (MARC0114654, *P* = 1.45E-10) at 126.83 Mb explaining 11.48% of phenotypic variance (Fig. 1d). We noted that two SNPs at 63.84 Mb (INRA0046679) and 63.98 Mb (MARC0049861) on SSC4 were significantly associated with C16:1n7/C16:0, C18:1n9/C18:0, UFA/SFA and MUFA/SFA. However, by applying linear homologous comparison via LASTZ^19^, we found that the genomic segment containing the two significant SNPs was inaccurately assembled, and the most likely location of this segment was in the vicinity of the 121.35 Mb region on SSC14 that was also significantly associated with the four metabolic traits mentioned above (Supplementary Fig. 2).

**Figure 2:**
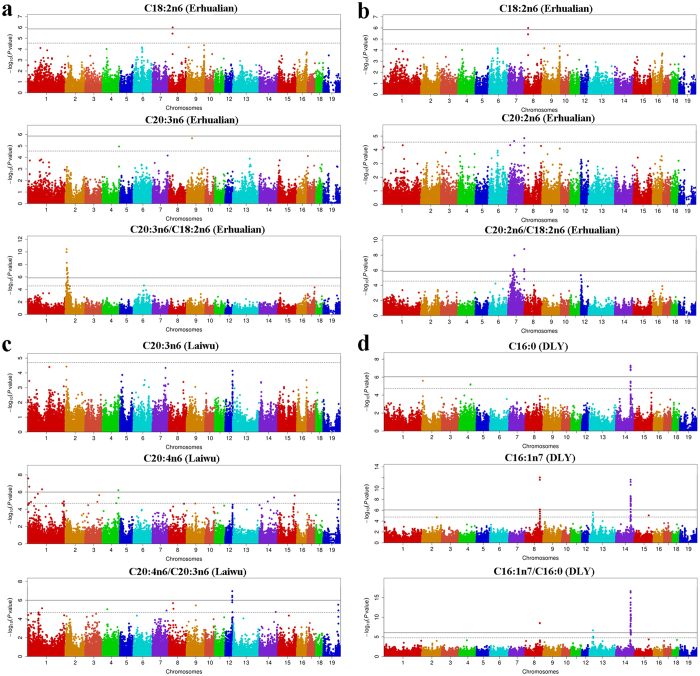
Manhattan plots of significant loci for fatty acid metabolic traits in the Erhualian, Laiwu and DLY populations. (**a**) GWAS results for C18:2n6, C20:3n6 and C20:3n6/C18:2n6 in the Erhualian population; (**b**) GWAS results for C18:2n6, C20:2n6 and C20:2n6/C18:2n6 in the Erhualian population; (**c**) GWAS results for C20:3n6, C20:4n6, and C20:4n6/C20:3n6 in the Laiwu population; (**d**) GWAS results for C16:0, C16:1n7 and C16:1n7/C16:0 in the DLY population; Negative log_10_ *P* values of the qualified SNPs (N) are plotted against their genomic positions. SNPs on different chromosomes are denoted by different colors. The solid line represents the genome-wide threshold (0.05/N). The dash line indicates the suggestive threshold (1/N).

In the Erhualian population, we identified seven genome-wide significant loci for 17 fatty acid metabolic traits on five chromosomes. By applying GWAS for fatty acid contents in this population, we have previously detected only one significant SNP (DIAS0001596) at 32.15 Mb on SSC8 for C18:2n6 content and did not identify any significant signals for C20:2n6 or C20:3n6 content^7^. In this study, a novel locus at 9.13–12.86 Mb on SSC2 containing 11 significant SNPs was detected for C20:3n6/C18:2n6 and C20:4n6/C20:3n6 (Fig. 2a). The top SNP (ASGA0008884, *P* = 3.55E-11) locates in the fifth intron of the *FADS2* (*fatty acid desaturase 2*) gene. This Erhualian-specific locus has not ever been reported in any association studies for fatty acids in pigs. Moreover, we identified two regions (36–56 Mb and 134.1–134.5 Mb) on SSC7 and one region (0.4–4 Mb) on SSC12 that were associated with C20:2n6/C18:2n6 at the genome-wide significant and suggestive levels, respectively (Fig. 2b). The 0.4–4 Mb region on SSC12 has been reported to be associated with multiple fatty acid contents, such as C14:0, C16:0, C16:1n7, C18:1n9, C20:1n9, but not with C18:2n6 or C20:2n6 content^7^. The locus at 134.1–134.5 Mb on SSC7 affects both fatty acid composition traits and metabolic traits in F_2_ and Erhualian pigs.

In the Laiwu population, we detected seven significant loci affecting 10 traits, which confirmed five previously reported QTL and revealed two novel loci. One novel chromosomal region at 57.80–61.49 Mb on SSC12 was associated with multiple metabolic traits, where six genome-wide SNPs for C20:4n6/C20:3n6, FattyTI, SFA and seven suggestive SNPs for PUFA/SFA, UFA/SFA, DBI and UI were found (Fig. 2c and Table 1). This pleiotropic locus was observed only in the Laiwu population. At 124.40–125.87 Mb on SSCX, we identified another novel locus associated with C20:2n6/C18:2n6 and C20:4n6/C20:2n6 (Table 1). This locus exists only in Laiwu pigs and have not been reported to associate with any fatty acid traits before this study.

In the DLY population, six chromosomal regions containing 114 SNPs were associated with 17 fatty acid metabolic traits. We have previously detected a significant locus (Top SNP: ASGA0066120, *P* = 2.42E-12) around 121.51 Mb on SSC14 for C16:1n7 content and a suggestive locus (28.52–31.47 Mb, *P* = 2.64E-06) on SSC13^7^. In this study, the association strength at the suggestive locus on SSC13 surpassed the genome-wide significant threshold for C16:1n7/C16:0 (*P* = 2.21E-07, Fig. 2d). The SSC13 locus is DLY-specific in the five populations. Moreover, the association strength at the SSC14 locus increased from 2.42E-12 for C16:1 to 2.24E-17 for C16:1n7/C16:0 (Fig. 2d).

### Meta-analysis of GWAS

**Figure 3:**
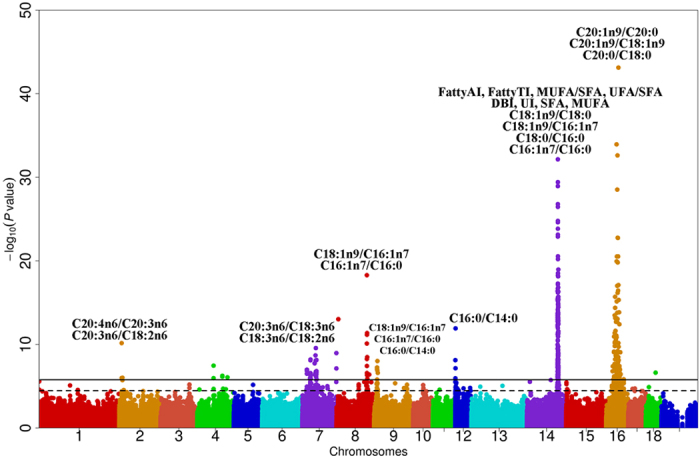
Manhattan plot of the GWAS meta-analysis across the five populations. The associated traits are indicated above the corresponding loci. Negative log_10_ *P* values of the qualified SNPs are plotted against their genomic positions. The solid line represents the genome-wide threshold (0.05/N). The dash line indicates the suggestive threshold (1/N). N stands for the common SNPs across the five populations.

We conducted a meta-analysis of GWAS for each fatty acid metabolic trait across the five populations. Genomic inflation factors (λ) in the meta-analysis were between 0.961 and 1.047, indicating that population stratification was properly adjusted. The meta-analysis integrates all association signals from the five populations. Therefore, when two or more populations show consistent association directions with target traits, the detection power for these traits will be improved by the meta-analysis of GWAS. By applying this analysis, we did not identify new loci but observed enhanced association strength at five loci, especially at the locus around 121 Mb on SSC14 and the locus around 43 Mb on SSC16 (Fig. 3). The most significant SNP for C18:1n9/C18:0 was ALGA0081091 (*P* = 1.19E-21) around 121 Mb on SSC14 in the DLY population. In the meta-analysis, the top SNP for C18:1n9/C18:0 was CASI0010164 (*P* = 7.07E-33) at 121.31 Mb on SSC14. Around 43 Mb on SSC16, the lead SNP for C20:0/C18:0 was DRGA0016155 (*P* = 7.69E-37) in the DLY population. We detected the same top SNP for the same trait at this locus with a much lower *P* value of 3.38E-52 (Fig. 3) in the meta-analysis. Two lead SNPs which were 12.69 Mb on SSC9 and 1.16 Mb on SSC12 showed consistent effects on C16:0/C14:0 across the five populations, conceivably resulting in an enhanced association signal in the meta-analysis (Fig. 3 and Supplementary Table 2). Moreover, more significant SNPs were detected at the 11–13 Mb region on SSC9 for C16:0/C14:0, C16:1n7/C16:0 and C18:1n9/C16:1n7 (Supplementary Table 2). The meta-analysis also characterized three new genome-wide significant loci for C16:1n7/C16:0 and C18:1n9/C16:1n7, which were located around 119.72 Mb on SSC8, 11.83 Mb on SSC9 and 121.30 Mb on SSC14 (Supplementary Table 2). Association signals at some significant loci, such as the Laiwu-specific locus at 57.80–61.49 Mb on SSC12 and the Erhualian-specific locus around 134 Mb on SSC7, were weakened or vanished in the meta-analysis (Supplementary Table 2). This could be due to population heterogeneity across the F_2_, Sutai, DLY, Laiwu and Erhualian populations.

### Conditional GWAS for pleiotropic, linked or common QTL

In the single-population GWAS, the significant loci that we identified were usually associated with more than one fatty acid metabolic trait. To test if there is only one pleiotropic QTL or more linked QTL at these loci, we conducted GWAS by including the most significant SNPs (shown in Table 1) as fixed effects in the model. When conditional on the effects of the top SNPs, most of association signals disappeared (Supplementary Fig. 3), supporting that common pleiotropic variants underlie these loci. For instance, the 119–123 Mb region on SSC14 was associated with multiple metabolic traits including C16:1n7/C16:0, C18:1n9/C18:0 and C18:0/C16:0. When we treated the top SNP (ALGA0081025 in F_2_, MARC0111695 in Sutai and ALGA0081091 in DLY) as a fixed effect, no association was evidenced for any traits around this region on SSC14, suggesting a real pleiotropic QTL in these populations. In contrast, after fixing the top population-specific SNPs (ASGA0033714 in F_2_, MARC0034834 in DLY, and INRA0025107 in Erhualian and INRA0026056 in Laiwu) on SSC7 in the GWAS model, association signals for all traits disappeared in F_2_ and DLY populations but retained in Laiwu and Erhualian pigs. In Laiwu pigs, another region around 28.06–37.39 Mb on this chromosome showed association with C16:0/C14:0, C16:1n7/C16:0, C18:1n9/C16:1n7, C20:1n9/C20:0 and FattyAI with the top SNP (DRGA0007448, *P* = 1.36E-09) at 31.62 Mb. In the Erhualian population, three SNPs were still significantly (*P* = 5.30E-09) associated with C18:1n9/C16:1n7, C20:1n9/C18:1n9, C20:1n9/C20:0 and C20:3n3/C18:3n3 even conditional on the effect of the top SNP. Moreover, when fixed the top SNPs (MARC0063090 and ASGA0099260) in Erhualian and Laiwu populations respectively, association signals of the 0.21–8.37 Mb region on SSC12 for C16:0/C14:0 disappeared in the Laiwu population, but a significant SNP (ASGA0052511, *P* = 6.13E-07) at 2.62 Mb together with seven suggestive variants in an adjacent region were still associated with C18:1n9/C16:1n7 in Erhualian pigs (Supplementary Fig. 3). This could be explained by the existence of multiple linked QTL in this region. Thus, higher-density markers or sequence data should be explored to clarify this issue in the near future.

Of note, the association signals for C20:0/C18:0 on SSC16 vanished in Erhualian and Laiwu populations when corrected for the effect of the top GWAS SNPs (ASGA0072949 in Erhualian and ASGA0072968 in Laiwu). These SNPs were located in an adjacent region of ~500 kb (Table 1). This leads us to assume that a common causal variant at the SSC16 locus is responsible for C20:0/C18:0 in the two populations. Given short linkage disequilibrium (LD) extents across Chinese pig populations^25^, the lead SNPs are most likely very close to the causal variant.

### Plausible candidate genes at significant loci

A list of strong candidate genes have been highlighted by our and other previous studies^4^,^5^,^6^,^7^,^21^,^22^, such as *SCD* on SSC14 for C18:0, *ELOVL5* on SSC7 for C20:1n9, *ELOVL6* on SSC8 for C16:1n7, *ELVOL7* on SSC16 for C20:0, *FASN* on SSC12 for C16:0, *ACSBG1* on SSC7 for C20:2n6 and *MTTP* on SSC8 for C14:1n5. By conducting GWAS on fatty acid metabolic traits, we herein proposed three novel candidate genes at the genome-wide significant loci.

At 9.13–12.86 Mb on SSC2, we detected a significant locus comprising 11 consecutive SNPs for C20:3n6/C18:2n6 and C20:4n6/C20:3n6 in Erhualian pigs. The top SNP (ASGA0008884) at this locus locates in the fifth intron of *fatty acid desaturase 2* (*FADS2*) gene. *FADS2* is a rate-limiting enzyme in the synthesis of long-chain polyunsaturated fatty acids through the introduction of double bond into *delta*-6 carbons in mammals. C20:3n6 and C18:2n6 are *delta*-6 unsaturated fatty acids. A nucleotide insertion in the transcriptional regulatory region of *FADS2* causes human fatty acid *delta*-6-desaturase deficiency^26^. Variants of *FADS2* are known to affect *omega*-6 and *omega*-3 milk fatty acids in cows^27^. Thus, *FADS2* is a promising candidate gene for the SSC2 locus.

At 57.80–61.49 Mb on SSC12, a significant locus for C20:4n6/C20:3n6, PUFA/SFA and SFA was found in the Laiwu population. The top SNP (ALGA0067099) locates between the *MYH13* and *MYH1* genes that have no obvious function related to fatty acid metabolism. However, comparative genomic analysis between human and pig shows that an interesting gene, *SREBF1* (*sterol regulatory element binding transcription factor 1*), is located in a homologous region (around 63.20 Mb) 1.8 Mb upstream of the locus. *SREBF1* is a transcription factor involved in fatty acid synthesis regulation. It initiates the transcription of more than 33 target genes that participate in synthesis of cholesterol, fatty acids, triglycerides, and phospholipids in mice^28^. So further studies are worthwhile for the *SREBF1* gene as a candidate for the SSC12 locus.

The 27–52 Mb region on SSC7 contains 150 significant SNPs associated with C20:4n6/C18:2n6, C20:4n6/C18:3 and C20:4n6/C20:3 in abdominal fat in the F_2_ population. The top SNP for C20:4n6/C18:2n6 and C20:4n6/C18:3 is ALGA0041153 at 48.90 Mb. *PLA2G7* that locates at 48.15 Mb on SSC7 is one of the arachidonic acid (C20:4n6) pathway members^29^ and could be a candidate gene for the three fatty acid metabolic traits.

### Gene networks and metabolic pathway

**Figure 4:**
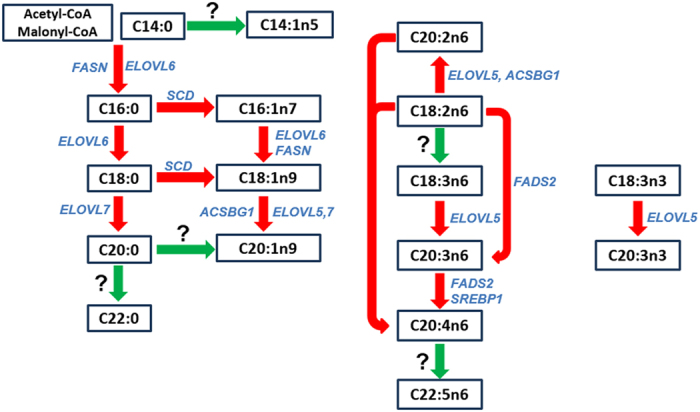
Graphical illustration of the potential fatty acid metabolism pathway and plausible candidate genes. The illustration shows the step-by-step synthesis of fatty acids. Significant loci have been identified for the ratios of substrates to products linked by red arrows. Plausible candidate genes that we highlighted for significant loci in this study are indicated alongside red arrows. For fatty acids linked by blue arrows, the loci for the metabolic ratios between these fatty acids remain unexplored.

We explored GeneMANIA^20^ to characterize enriched functional processes and gene networks involving the candidate genes highlighted for both significant (Table 1) and suggestive (Table 2, also see below) loci. Most of candidate genes were enriched in biological processes related to fatty acid metabolism, such as fatty acid metabolic process (FDR = 1.17E-18), acylglycerol and neutral lipid metabolic process (FDR = 3.16E-16), triglyceride metabolic process and long-chain fatty-acyl-CoA metabolic process (FDR = 6.68E-15), fatty-acyl-CoA metabolic (FDR = 1.46E-14) and fatty-acyl-CoA biosynthesis process (FDR = 9.02E-14, Supplementary Table 3). A total of 404 links were observed in the gene networks related to fatty acid metabolism, which included additional 19 genes that were not identified in this study (Supplementary Fig. 4, Supplementary Tables 3 and 4). The 19 genes are also functionally related to fatty acid metabolism. Moreover, many genes co-occurred in multiple networks. For example, the *ELOVL* family genes and *FADS* family genes appeared in many pathways, including fatty acid metabolic and biosynthetic process, carboxylic acid biosynthetic process and long-chain fatty acid metabolic process. This emphasizes the importance of the *ELOVL* and *FADS* family genes as key driver enzymes in fatty acid elongation and desaturation. Altogether, the interactive analysis of candidate genes gives us a visually understanding of metabolic pathway for fatty acid composition (Fig. 4).

### Suggestive loci

The Bonferroni-corrected threshold is too stringent to detect a significant locus with moderate or small effects^30^. Thus, we defined 12 suggestive loci on nine chromosomes (Table 2) for the tested traits at a less strict level of 1/N, where N represents the number of qualified SNPs in each population. To our knowledge, many of suggestive loci are reported for the first time, such as the locus at 107.47–114.96 Mb on SSC2 for C20:4n6/C18:2n6 in the Sutai population, the locus at 17.32–17.92 Mb on SSC5 for C20:0/C18:0 in the Laiwu population and the locus at 5.92–9.14 Mb on SSC6 for C20:2/C18:2n6 in the F_2_ population. At five suggestive loci (107.47–114.96 Mb on SSC2, 5.92–9.14 Mb on SSC6, 65.19–71.01 Mb on SSC6, 28.85–33.74 Mb on SSC8 and 11.48–14.51 Mb on SSC9), we found functionally plausible candidate genes, including *PAM*, *AADACL3*, *TBC1D1*, and *DGAT2*. These genes have been associated with fatty acid contents in previous studies^31^,^32^,^33^,^34^ and warrant further investigations.

## Discussion

### GWAS on metabolic traits identifies novel loci for fatty acid composition

To our knowledge, although QTL for fatty acid indices have been reported in previous studies^4^,^6^,^21^,^35^,^36^, this is the first GWAS on fatty acid metabolic traits in multiple diverse pig populations. In total, we identified 867 genome-wide significant SNPs, 97.25% of which locate in noncoding regions, indicative of complex regulatory mechanisms for fatty acid metabolism. Of note, our findings not only replicated the previously reported loci for fatty acid composition, but also detected four novel significant loci, including the SSC2 locus (9.13–12.86 Mb) for C20:3n6/C18:2n6 in Erhualian, the SSC12 (57.80–61.49 Mb) locus for C20:4n6/C20:3n6 in Laiwu, the SSC13 locus (28.52–31.47 Mb) for C16:1n7/C16:0 in DLY and the SSCX locus (124.40–125.87 Mb) for C20:4n6/C20:2n6 in Laiwu. The four loci are population specific loci (Figs 1 and 2). This supports the assumption that GWAS on metabolic ratios can increase the power of GWAS compared to that of association of individual metabolites. As pointed by Petersen *et al.*^37^, if two metabolites are a product-substrate of an enzymatic reaction, then ratios between their concentrations are potential proxies of the enzymatic reaction rate. When the SNPs in an enzyme-coding gene show much stronger (ten times or more) association with the metabolic ratio than with two individual metabolites in GWAS, then the enzyme is likely the one responsible for conversion of substrate into product, even if the molecular function of that enzyme was not already known. So using metabolic ratios as quantitative traits for GWAS may allow us to deduce new enzymatic activities. For example, variants proximal to the *FADS2* gene are significantly associated with the ratio of C20:4n6/C20:3n6. The findings enable us to deduce that *FADS2* may catalyze the conversion of C20:4n6 from C20:3n6, which remained unknown before this study.

### GWAS on metabolic traits increases association strength at several significant loci

In this study, GWAS on fatty acid metabolic traits increased association strength at several significant loci for fatty acid contents. For example, we have previously identified a significant locus on SSC16 for C20:0 content across the five populations. The top SNPs at 43.53 Mb (DRGA0016155, *P* = 4.32E-31), 34.71 Mb (ASGA0072949, *P* = 6.97E-13) and 45.31 Mb (DRGA0016169, *P* = 7.41E-14) on SSC16 explained 31.79%, 12.07% and 25.50% phenotypic variance in the DLY, Erhualian and Laiwu populations, respectively. Interestingly, by conducting the GWAS for C20:0/C18:0, more SNPs on SSC16 (N = 62, 51 and 61) were detected in the three populations. The top SNPs at 43.53 Mb (DRGA0016155, *P* = 7.69E-37), 34.71 Mb (ASGA0072949, *P* = 3.92E-18) and 35.19 Mb (ASGA0072968, *P* = 3.50E-17) explained 39.97%, 20.21% and 31.16% of phenotypic variance in these populations, respectively. This again illustrates that GWAS on fatty acid metabolic traits could result in more associated SNPs with stronger association strength explaining more phenotypic variance. This is consistent with previous reports that GWAS on metabolic ratios as intermediate phenotypes can reduce the variance of the dataset and yield enhanced association statistics^10^,^37^.

### The deduced metabolic pathway of fatty acids advances our understanding of the genetic mechanism of fatty acid composition

GWAS on fatty acid metabolic traits provide us important clues to deduce the fatty acid metabolic pathway (Fig. 4), in which different genes are responsible for the step-by-step synthesis of fatty acids. We noted that several loci were population specific, indicating that variants in some genes in the pathway may exert their effects on fatty acid metabolism in a population-specific manner. For example, the locus at 134.54 Mb on SSC7 containing *ELOVL5* was detected for C20:1n9/C18:1n9 and C20:2n6/C18:2n6 in F_2_ and Erhulian pigs. So the *ELVOL5* is more likely to elongate C18:1n9 and C18:2n6 acyl-CoAs to C20:1n9 and C20:2n6 acyl-CoAs in the two populations. In Erhualian population, the same locus was also associated with C20:3n3/C18:3n3; thus, *ELOVL5* could specifically elongate C18:3n3 acyl-CoA to C20:3n3 acyl-CoA in this population. Some genes appeared to exhibit pleiotropic effects in multiple metabolic steps. For example, the *ELOVL6* gene could not only catalyzes two carbons to C16:0 acyl-CoA to synthesize C18:0 acyl-CoA, but also mediates the synthesis of C18:1n9 acyl-CoA from C16:1n7 acyl-CoA. This is in conflict with the previous assumption that *ELOVL6* is only involved in the elongation of C12-16 saturated fatty acid to C18 saturated fatty acid in mammals^38^. Although the deduced metabolic pathway allows us to better understand substrate-product relationships and the genetic mechanism of fatty acid compositions, more experimental assays are needed to validate the deduced pathway.

### Biological, mediate and spurious pleiotropy

Pleiotropy widely exists in species. In the human genome, 16.9% of genes and 4.6% of SNPs show pleiotropic effects^39^. Generally, there are three kinds of pleiotropy: biological, mediate and spurious pleiotropy^40^. We detected a significant locus at 121.35 Mb on SSC14 for C16:1n7/C16:0 and C18:1n9/C18:0 in the F_2_ and DLY populations. Interestingly, the candidate gene *SCD* within this region catalyzes conversion of both C16:0 and C18:0 to C16:1n7 and C18:1n9. Thus, it is likely that a common causative mutation with the *SCD* gene underlie the pleiotropic locus on SSC14. The locus at 27.45–80.45 Mb on SSC7 containing 210 SNPs influences 12 metabolic traits in abdominal fat and *longissimus dorsi* muscle in the F_2_ population (Table 1). We found high LD extents within the chromosome region harboring these 210 SNPs in this population (Supplementary Fig. 5a). Thus, it is conceivable to observe that the association signals of all these 210 SNPs disappeared when conditional on the effect of the top SNP (ASGA0033714) at this locus by treating it as a fixed effect in the GWAS model (Supplementary Fig. 3). This observation support the locus as a biological pleiotropy. However, we cannot rule out the possibility that different causative mutations having strong LD with the top SNP may co-localize at this locus. We note that the top SNP has moderate LD (*r* ^2^ < 0.5) with these 210 SNPs (Supplementary Fig. 5a), indicating that the causative mutation underlying the SSC7 locus is likely located in the vicinity of the top SNP. The pleiotropic QTL with a beneficial allelic effect on more than one trait warrant further investigations to characterize the underlying mutations, which would enable us to develop novel molecular breeding tools for optimizing fatty acid compositions in pork.

The 0.4–8.29 Mb region on SSC12 was significantly associated with seven metabolic traits in the Erhualian population. When the top SNP (MARC0063090) was fixed in the GWAS model, the association signal for C18:1n9/C16:1n7 still surpassed the genome-wide significant threshold with the top SNP (ASGA0052511) at 2.62 Mb on this chromosome (Supplementary Fig. 3). We found that the LD between the two top SNPs (MARC0063090 and ASGA0052511) is relatively low (*r*^2^ = 0.38, Supplementary Fig. 5b). This supports our assumption that the locus is a spurious pleiotropic one and different causative variants are in LD with the lead SNPs.

It is difficult to distinguish mediate pleiotropy from biological and spurious pleiotropy. In this study, the locus at 9.13–12.86 Mb on SSC2 was associated with C20:3n6/C18:2n6 and C20:4n6/C20:3n6 in Erhualian pigs. C20:3n6 can be converted to C20:4n6 by fatty acid *delta*-desaturase, while C18:2n6 cannot be directly converted to C20:3n6, which must be converted to C18:3n6 by fatty acid *delta*-desaturase and then transformed to C20:3n6 by *ELOVL5*^41^. By treating C20:3n6/C18:2n6 as a fixed effect in GWAS model, the top SNP changed from ASGA0008884 (*P* = 3.54E-11) to ALGA0011877 (*P* = 7.53E-07). Thus, the locus may be a mediate pleiotropic locus and C20:3n6/C18:2n6 is likely an intermediate phenotype.

## Additional Information

**How to cite this article**: Zhang, W. *et al.* Genome-wide association studies for fatty acid metabolic traits in five divergent pig populations. *Sci. Rep.* **6**, 24718; doi: 10.1038/srep24718 (2016).

## Supplementary Material

Supplementary Information

## Figures and Tables

**Table 1:** Summary of significant loci for fatty acid metabolic indices in five pig populations.

Chr	Trait	Pop	Nsnp	Range of SNP (Mb)	Top SNP	Position (bp)	*P*-Value	Candidate genes
2	C20:3n6/C18:2n6, C20:4n6/C20:3n6	Erhualian	11	9.13–12.86	ASGA0008884	9139348	3.55E-11	*FADS2*
7	A20:1n9/A18:1n9, A20:1n9/A20:0, A20:2n6/A18:2n6, A20:4n6/A18:2n6, A20:4n6/A18:3, A20:4n6/A20:3, A22:5/A20:4n6, AMUFA, APUFA, APUFA/SFA, C20:1n9/C18:1n9, C20:4n6/C18:3,	F_2_	210	27.45–80.45	ASGA0033714	53659203	2.31E-13	*PLA2G7*
	A20:1n9/A18:1n9, A20:1n9/A20:0, A20:2n6/A18:2n6, A20:4n6/A18:2n6, A20:4n6/A18:3, C20:1/C18:1n9	F_2_	4	134.27–134.68	MARC0060950	134313767	1.52E-12	*ELOVL5*
	C20:2n6/C18:2n6	DLY	3	52.80–53.56	MARC0034834	53102034	5.42E-08	*ACSBG1*
	C20:1n9/C18:1n9, C20:1n9/C20:0, C20:2n6/C18:2n6, C20:3n3/C18:3n3, C20:3n6/C18:3n6,	Erhualian	42	32.23–59.70	INRA0025107	41743168	1.03E-09	
	C20:1n9/C18:1n9, C20:1n9/C20:0, C20:2n6/C18:2n6, C20:3n3/C18:3n3,	Erhualian	6	134.15–134.54	ALGA0114746	134540651	2.84E-20	
	C16:1n7/C16:0, C18:1n9/C16:1n7, C20:1n9/C18:1n9, C20:1n9/C20:0, C20:2n6/C18:2n6, C20:3n3/C18:3n3	Laiwu	70	28.07–76.29	INRA0026056	58234493	1.30E-16	
8	A18:0/A16:0, A18:1n9/A16:1n7, C16:0/C14:0, C18:1n9/C16:1n7	F_2_	18	119.42–129.56	MARC0114654	126831850	1.43E-10	*ELOVL6*
	C14:1n5/C14:0, C18:1n9/C16:1n7	Erhualian	2	127.07–138.50	DIAS0004322	138501500	6.43E-10	*MTTP*
	C16:1n7/C16:0, C18:0/C16:0, C18:1n9/C16:1n7	DLY	8	119.72–121.47	H3GA0025321	119887525	6.40E-17	
9	C16:1n7/C14:0	DLY	1	14.21	MARC0100725	14217867	4.73E-08	
12	C14:1n5/C14:0, C16:0/C14:0, C16:1n7/C16:0, C18:1n9/C16:1n7, DBI, FattyAI, UI	Erhualian	10	0.4–8.29	MARC0063090	1779278	2.09E-13	*FASN*
	C16:0/C14:0	Laiwu	8	0.2–8.37	ASGA0099260	248014	2.06E-10	
	C20:4n6/C20:3n6, PUFA/SFA, SFA,	Laiwu	6	57.80–61.49	ALGA0067099	57950908	1.08E-07	*SREBF1*
13	C16:1n7/C16:0	DLY	1	28.52	ASGA0104338	28524131	2.21E-07	
14	C16:1n7/C16:0, C18:0/C16:0, C18:1n9/C18:0, MUFA/SFA, UFA/SFA	F_2_	23	119.51–123.40	ALGA0081025	119955763	3.47E-10	*SCD*
	C18:0/C16:0, C18:1n9/C18:0, FattyTI, SFA, UFA/SFA	Sutai	12	116.35–119.96	MARC0111695	116694325	1.59E-09	
	C16:1n7/C16:0, C18:0/C16:0, C18:1n9/C16:1n7, C18:1n9/C18:0, DBI, FattyAI, FattyTI, MUFA, MUFA/SFA, SFA, UFA/SFA, UI	DLY	38	120.21–123.29	ALGA0081091	120986865	1.19E-21	
16	A20:0/A18:0, A20:1n9/A20:0, C20:0/C18:0, C20:1n9/C20:0	F_2_	162	21.23–63.72	ALGA0090423	41393886	6.51E-24	*ELOVL7*
	C20:0/C18:0	Sutai	79	21.71–50.25	MARC0073781	28101697	2.79E-09	
	C20:0/C18:0, C20:1n9/C20:0, C20:1n9/C18:1n9	DLY	62	35.24–49.17	DRGA0016155	43534471	7.69E-37	
	C20:0/C18:0, C20:1n9/C20:0	Erhualian	51	26.89–55.96	ASGA0072949	34715842	3.92E-18	
	C20:0/C18:0	Laiwu	61	24.67–62.24	ASGA0072968	35190026	3.50E-17	
X	C20:2n6/C18:2n6, C20:4n6/C20:2n6	Laiwu	2	124.40–125.87	H3GA0052009	125876415	2.69E-07	

Chr: chromosome; Pop, population; F_2_, the White Duroc × Erhualian F_2_ intercross; DLY, Duroc × (Landrace × Yorkshire) hybrid pigs; Nsnp, the total number of significant SNPs associated with the traits; Top SNP, the most significantly associated SNP for a given trait; Position, genomic position of the top SNP on the Sscrofa 10.2 pig genome assembly; *P*-value, the *P*-value of the top SNP; *P*-values were calculated by the use of the GenABEL package in R.

**Table 2:** Summary of suggestive loci for fatty acid metabolic indices in five pig populations.

Chr	Trait	Pop	Nsnp	Range of SNP (Mb)	Top SNP	Position (bp)	*P*-value	Candidate genes
2	C20:4n6/C18:2n6, C20:4n6/C20:2n6	Sutai	10	107.47–114.96	MARC0080651	109657735	1.30E-06	*PAM*
4	A_UI, A_DBI, APUFA/SFA, APUFA	F_2_	3	89.00–91.69	ALGA0026229	89038458	7.02E-06	*PBX1*
5	C20:0/C18:0	Laiwu	8	17.32–17.92	H3GA0015868	17786755	3.71E-06	*SCN8A*
6	C20:2/C18:2n6	F_2_	7	5.92–9.14	M1GA0026870	8453407	1.78E-06	*HSDL1*
	ACL, N6/N3, C16:1n7/C16:0, C18:0/C16:0, C18:1n9/C16:1n7	Laiwu	4	65.19–71.01	ASGA0106005	71011913	1.11E-06	*AADACL3*
8	C16:1n7/C16:0, C18:1n9/C16:1n7, N3, N6, PUFA, PUFA/MUFA	Erhualian	4	28.85–33.74	H3GA0056028	33740711	1.87E-06	*TBC1D1*
	C20:1n9/C20:0, C20:3n6/C18:3n6, MUFA	DLY	4	17.53–18.14	ASGA0037971	17530855	4.99E-06	
9	C16:0/C14:0, C16:1n7/C16:0, C18:1n9/C16:1n7	Laiwu	6	11.48–14.51	ALGA0051583	14447589	2.28E-06	*DGAT2*
	C16:0/C14:0, C20:1n9/C20:0	DLY	2	11.34–14.30	MARC0046646	14306925	6.20E-06	
10	C16:0/C14:0, C20:1n9/C20:0	F_2_	2	68.62–70.25	ASGA0048928	70250117	1.29E-06	
	C20:4n6/C20:2n6	Laiwu	2	69.82–70.55	M1GA0014342	69820079	2.49E-06	
13	C14:1n5/C14:0	Sutai	3	71.04–72.23	DRGA0012717	72216697	1.55E-08	
15	C20:4n6/C18:2n6, C20:4n6/C20:2n6	Laiwu	6	13.26–17.10	CASI0010463	15140520	6.82E-06	
	C20:4n6/C20:2n6, N6/N3	Laiwu	3	127.72–130.59	ALGA0086940	130249305	1.17E-06	
	C14:1n5/C14:0, C20:1n9/C20:0, C20:1n9/C18:1n9, C20:4n6/C20:2n6	Laiwu	11	144.54–147.38	H3GA0045535	147384455	3.03E-06	

Chr: chromosome; Pop, population; Nsnp, the total number of significant SNPs associated with the traits; F_2_, the White Duroc × Erhualian F_2_ intercross; DLY, Duroc × (Landrace × Yorkshire) hybrid pigs; Top SNP, the most significantly associated SNP for a given trait; Position, genomic position of the top SNP on the Sscrofa 10.2 pig genome assembly; *P*-value, the *P*-value of the top SNP; *P*-values were calculated by the use of the GenABEL package in R.
