# Supplementary material for: Genome-wide association studies for fatty acid metabolic traits in five divergent pig populations
Source: Sci Rep. 2016 Apr 21;6:24718. doi: 10.1038/srep24718 (PMC4838829; doi:10.1038/srep24718)
Supplement: Supplementary Information [file srep24718-s1.pdf]

Supplementary Information of:

Genome-wide association studies for fatty acid metabolic traits in five divergent pig populations

Wanchang Zhang[1,2], Bin Yang[1,2], Junjie Zhang[1], Leilei Cui[1], Junwu Ma[1], Congying Chen[1], Huashui Ai[1], Shijun Xiao[1], Jun Ren[1,3] & Lusheng Huang[1]

[1]State Key Laboratory of Pig Genetic Improvement and Production Technology, Jiangxi Agricultural University, Nanchang, 330045, P.R. China

[2]These authors contributed equally to this work.

[3]Correspondence should be addressed to J.R. (email: renjunjxau@hotmail.com)

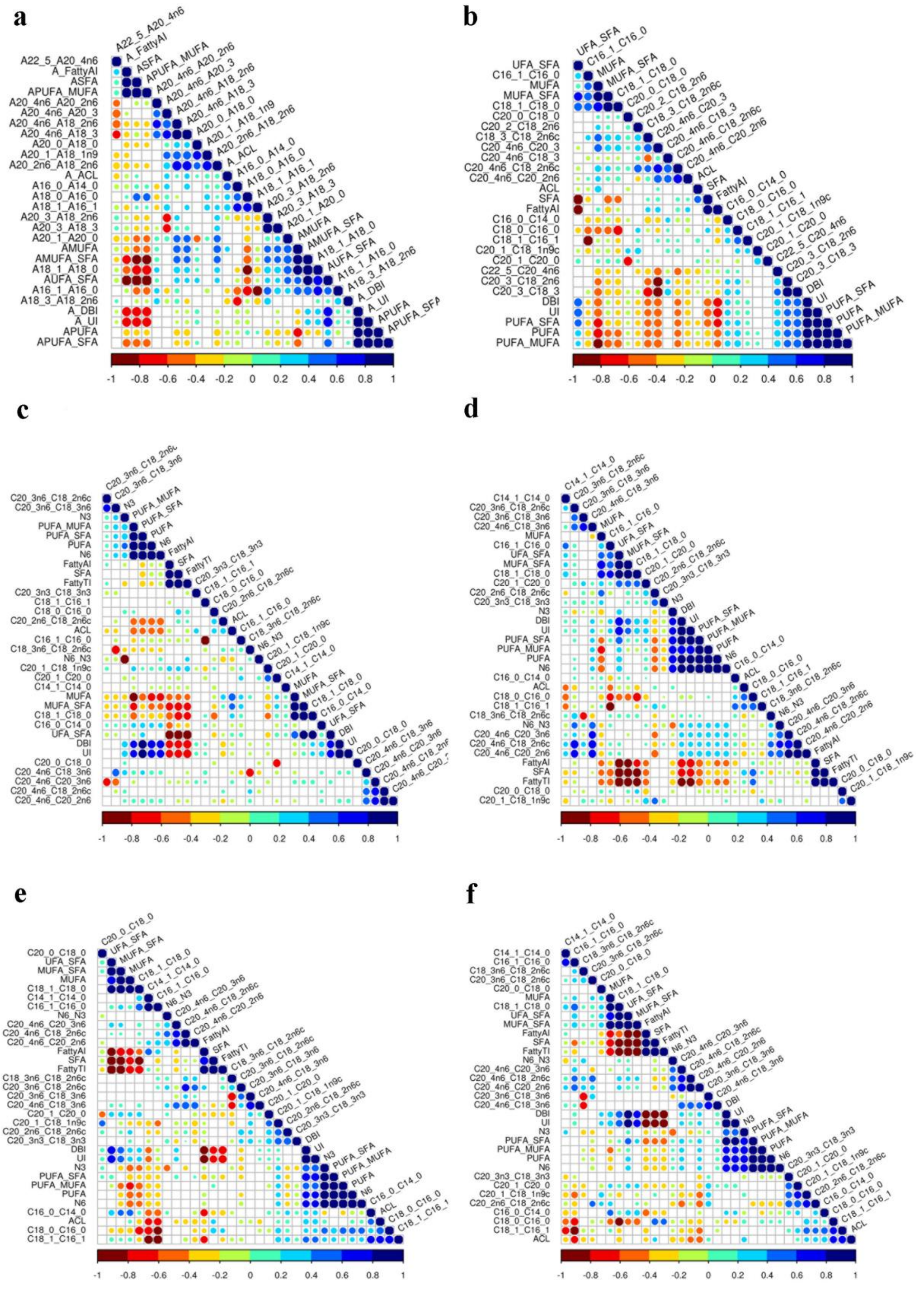
a
A22_5_A20_4n6
A_FattyAI
ASFA
APUFA_MUFA
A20_4n6_A20_2n6
A20_4n6_A20_3
A20_4n6_A18_2n6
A20_4n6_A18_3
A20_0_A18_0
A20_1_A18_1n9
A20_2n6_A18_2n6
A_ACL
A16_0_A14_0
A18_0_A16_0
A18_1_A16_1
A20_3_A18_2n6
A20_3_A18_3
A20_1_A20_0
AMUFA
AMUFA_SFA
A18_1_A18_0
AUFA_SFA
A16_1_A16_0
A18_3_A18_2n6
A_DBI
A_UI
APUFA
APUFA_SFA
-1 -0.8 -0.6 -0.4 -0.2 0 0.2 0.4 0.6 0.8 1
b
UFA_SFA
C16_1_C16_0
MUFA
MUFA_SFA
C18_1_C18_0
C20_0_C18_0
C20_2_C18_2n6
C18_3_C18_2n6c
C20_4n6_C20_3
C20_4n6_C18_3
C20_4n6_C18_2n6c
C20_4n6_C20_2n6
ACL
SFA
FattyAI
C16_0_C14_0
C18_0_C16_0
C18_1_C16_1
C20_1_C18_1n9c
C20_1_C20_0
C22_5_C20_4n6
C20_3_C18_2n6
C20_3_C18_3
DBI
UI
PUFA_SFA
PUFA
PUFA_MUFA
-1 -0.8 -0.6 -0.4 -0.2 0 0.2 0.4 0.6 0.8 1
c
C20_3n6_C18_2n6c
C20_3n6_C18_3n6
N3
PUFA_MUFA
PUFA_SFA
PUFA
N6
FattyAI
SFA
FattyTI
C20_3n3_C18_3n3
C18_1_C16_1
C18_0_C16_0
C20_2n6_C18_2n6c
ACL
C16_1_C16_0
C18_3n6_C18_2n6c
N6_N3
C20_1_C18_1n9c
C20_1_C20_0
C14_1_C14_0
MUFA
MUFA_SFA
C18_1_C18_0
C16_0_C14_0
UFA_SFA
DBI
UI
C20_0_C18_0
C20_4n6_C18_3n6
C20_4n6_C20_3n6
C20_4n6_C18_2n6c
C20_4n6_C20_2n6
-1 -0.8 -0.6 -0.4 -0.2 0 0.2 0.4 0.6 0.8 1
d
C14_1_C14_0
C20_3n6_C18_2n6c
C20_3n6_C18_3n6
C20_4n6_C18_3n6
MUFA
C16_1_C16_0
UFA_SFA
MUFA_SFA
C18_1_C18_0
C20_1_C20_0
C20_2n6_C18_2n6c
C20_3n3_C18_3n3
N3
DBI
UI
PUFA_SFA
PUFA_MUFA
PUFA
N6
C16_0_C14_0
ACL
C18_0_C16_0
C18_1_C16_1
C18_3n6_C18_2n6c
N6_N3
C20_4n6_C20_3n6
C20_4n6_C18_2n6c
C20_4n6_C20_2n6
FattyAI
SFA
FattyTI
C20_0_C18_0
C20_1_C18_1n9c
-1 -0.8 -0.6 -0.4 -0.2 0 0.2 0.4 0.6 0.8 1
e
C20_0_C18_0
UFA_SFA
MUFA_SFA
MUFA
C18_1_C18_0
C14_1_C14_0
C16_1_C16_0
N6_N3
C20_4n6_C20_3n6
C20_4n6_C18_2n6c
C20_4n6_C20_2n6
FattyAI
SFA
FattyTI
C18_3n6_C18_2n6c
C20_3n6_C18_2n6c
C20_3n6_C18_3n6
C20_4n6_C18_3n6
C20_1_C20_0
C20_1_C18_1n9c
C20_2n6_C18_2n6c
C20_3n3_C18_3n3
DBI
UI
N3
PUFA_SFA
PUFA_MUFA
PUFA
N6
C16_0_C14_0
ACL
C18_0_C16_0
C18_1_C16_1
-1 -0.8 -0.6 -0.4 -0.2 0 0.2 0.4 0.6 0.8 1
f
C14_1_C14_0
C16_1_C16_0
C18_3n6_C18_2n6c
C20_3n6_C18_2n6c
C20_0_C18_0
MUFA
C18_1_C18_0
UFA_SFA
MUFA_SFA
FattyAI
SFA
FattyTI
N6_N3
C20_4n6_C20_3n6
C20_4n6_C18_2n6c
C20_4n6_C20_2n6
C20_3n6_C18_3n6
C20_4n6_C18_3n6
DBI
UI
N3
PUFA_SFA
PUFA_MUFA
PUFA
N6
C20_3n3_C18_3n3
C20_1_C20_0
C20_1_C18_1n9c
C20_2n6_C18_2n6c
C16_0_C14_0
C18_0_C16_0
C18_1_C16_1
ACL
-1 -0.8 -0.6 -0.4 -0.2 0 0.2 0.4 0.6 0.8 1

Supplementary Fig. 1

Correlation matrix plots between fatty acid metabolic traits in five pig populations, including the F_2 (abdominal fat, a), F_2 (muscle, b), Sutai (c), DLY (d), Erhualian (e), and Laiwu (f) populations. Correlation coefficients between each pair of traits with $P < 0.05$ are plotted as dots. Their sizes and colors represent the degree and direction. Blue is positive and red is negative. The larger the dot, the stronger the correlation coefficient. All diagonal elements are marked above the corresponding column of the traits.

Zhang *et al.*, Supplementary Fig. 1

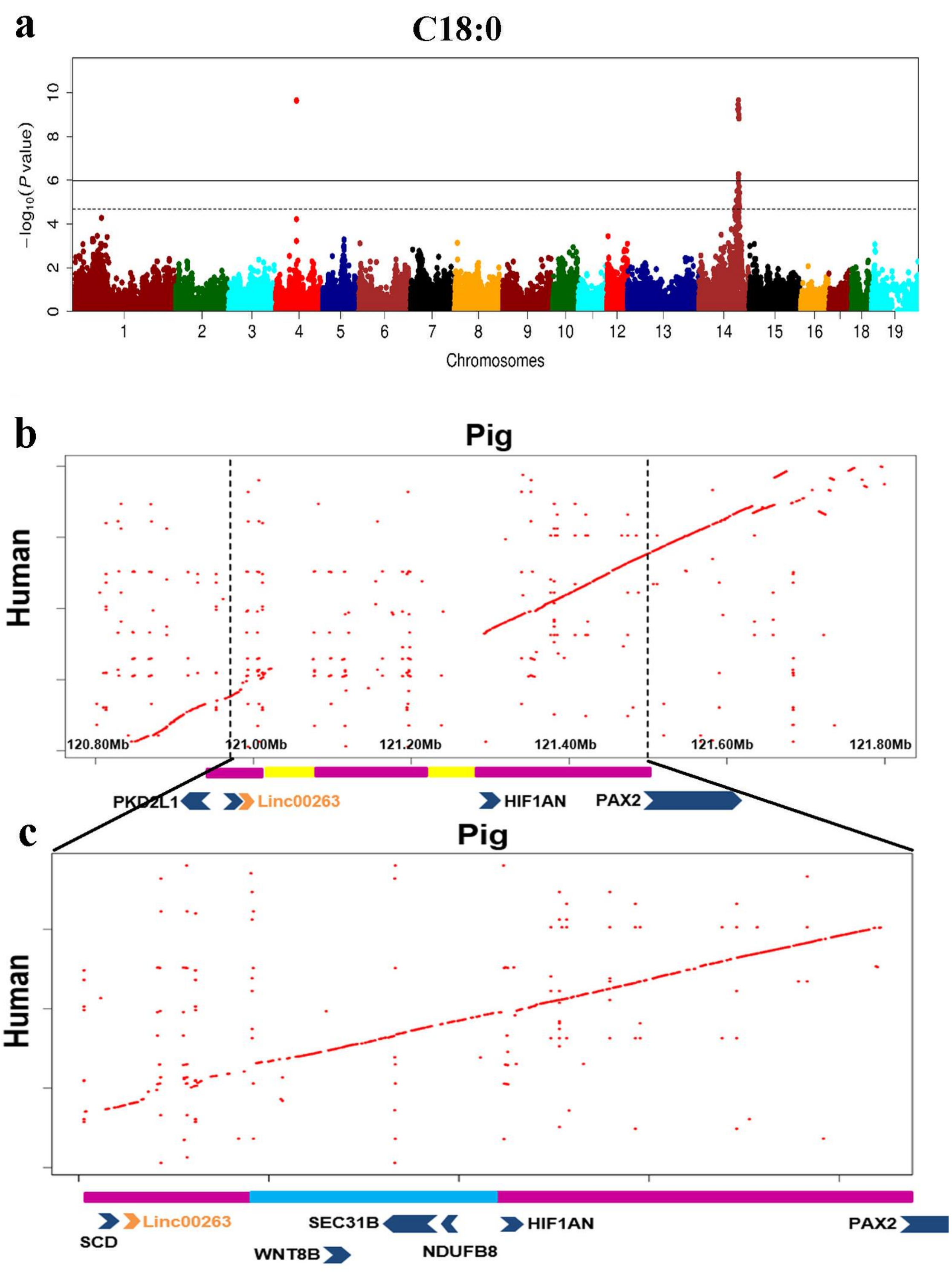
a
C18:0
−log10(P value)
Chromosomes
b
Pig
Human
120.80Mb
121.00Mb
121.20Mb
121.40Mb
121.60Mb
121.80Mb
PKD2L1
Linc00263
HIF1AN
PAX2
c
Pig
Human
SCD
Linc00263
SEC31B
NDUFB8
WNT8B
HIF1AN
PAX2

Supplementary Fig. 2

Human-pig comparative genomic analysis of the QTL region on SSC14 that is significantly associated with C16:1n7/C16:0 and C18:1n9/C18:0 in the F_2, Sutai and DLY populations. (**a**) Manhattan plot of GWAS results for C18:1 content in the F_2 population. A SNP on chromosome 4 shows association strength similar to the region around 121 Mb on chromosome 14. (**b**) The purple line represents a 580 kb QTL region on chromosome 14 in pig; the yellow lines indicates two sequence gaps. (**c**) After realignment with sequence of pig chromosome 4 (blue line), the reassembled sequence of the SSC14 QTL shows better homology with human genome sequence.

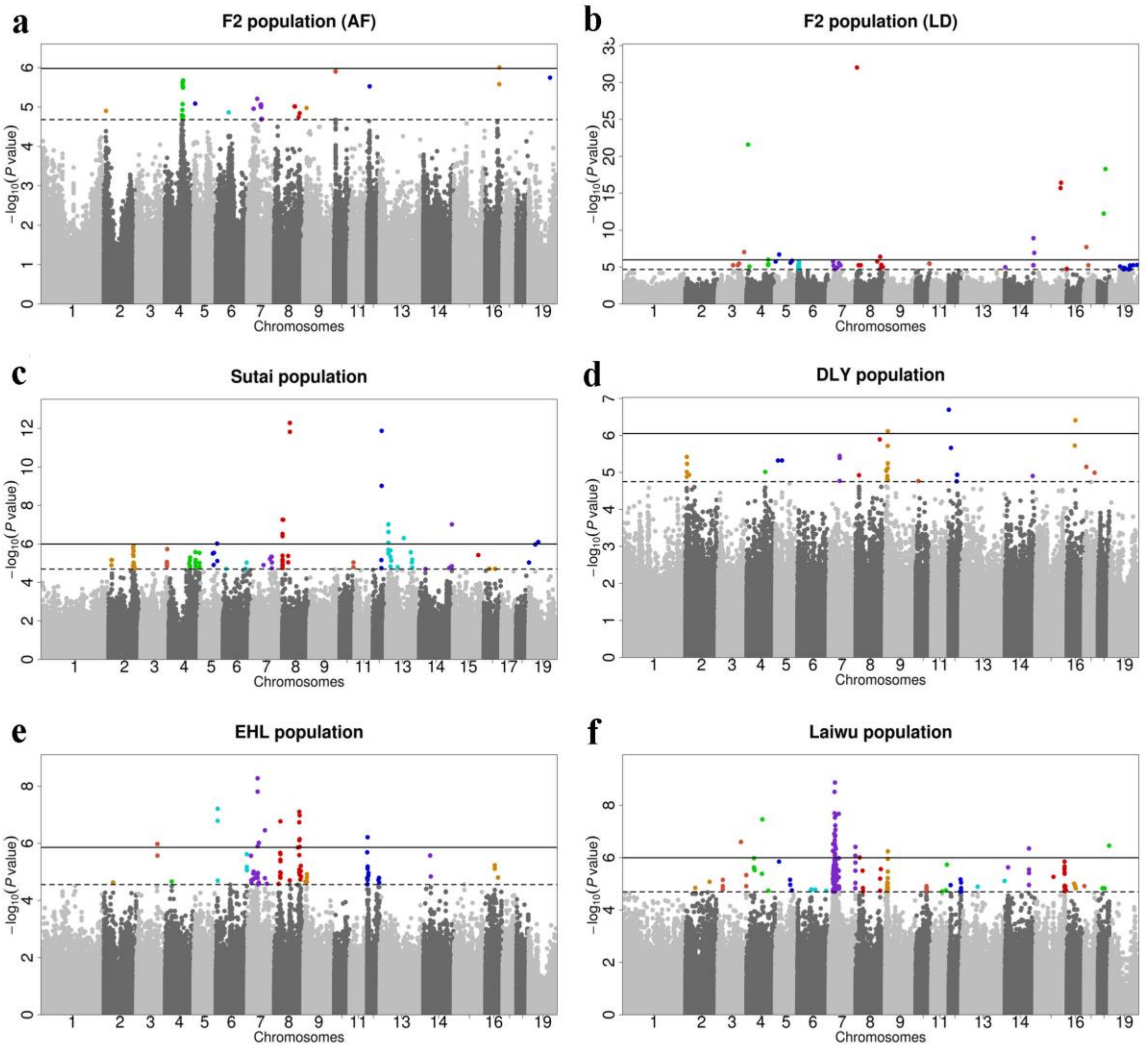

Supplementary Fig. 3

Manhattan plots of conditional GWAS results across five populations. Conditional GWAS results are shown for the F_2 (abdominal fat, **a**), F_2 (muscle, **b**), Sutai (**c**), DLY (**d**), Erhualian (**e**) and Laiwu (**f**) populations. The solid line represents the genome-wide threshold (0.05/N). The dash line indicates the suggestive threshold (1/N). N represents the number of SNP that passed quality control. Significant signals surpassing the suggestive threshold on different chromosomes are plotted with different colors.

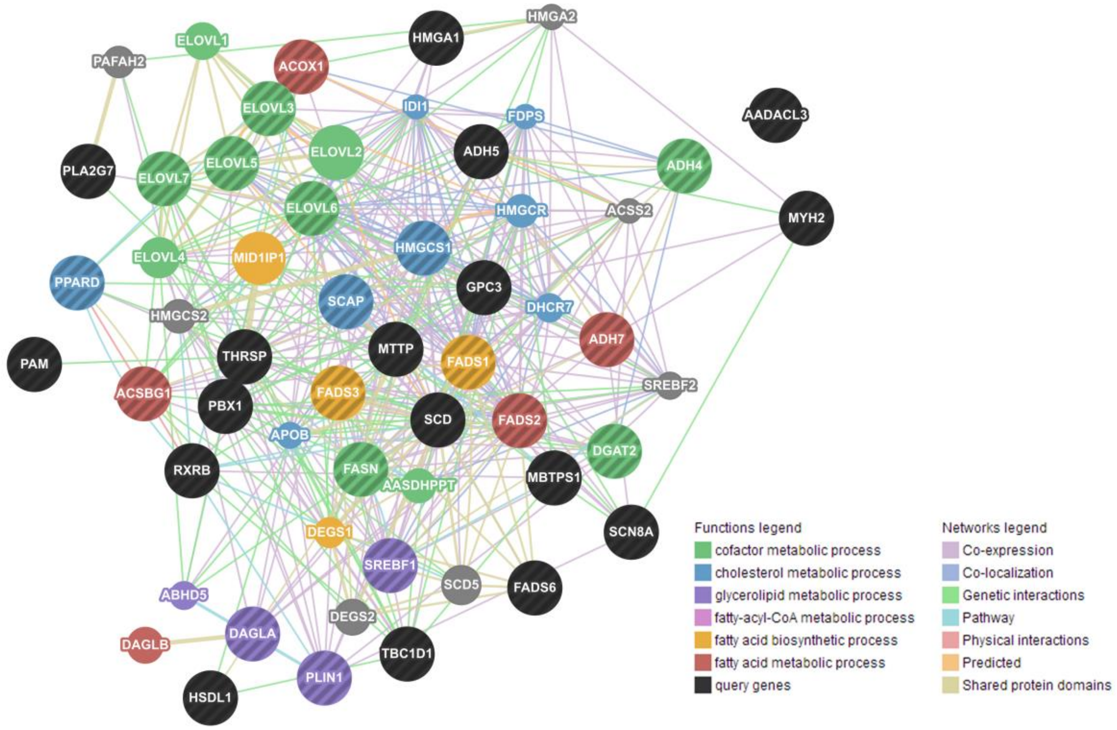

Supplementary Fig. 4

Gene networks and functional pathways for candidate genes highlighted in this study. GeneMANIA was explored to construct networks of candidate genes. Nodes represent candidate genes and links indicate different interactive connections. Links of different colors are different interactive networks.

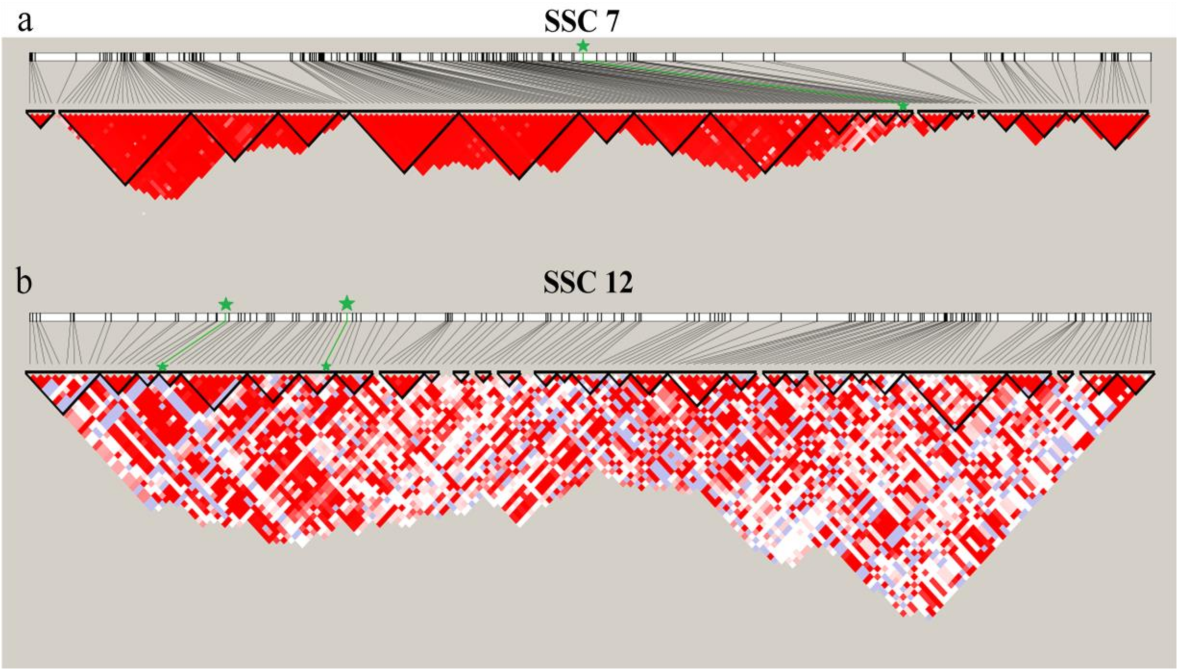

Supplementary Fig. 5

Plots of linkage disequilibrium (LD) pattern at two significant loci detected in this study. (**a**) The LD extent plot for the 27.45-80.45 Mb region on SSC7. The green star indicates the location of the top SNP (ASGA0033714) for fatty acid metabolic traits. (**b**) The LD extent plot for the 0.4 - 8.29 Mb region on SSC12. The green stars indicate the locations of the two top SNPs (left: MARC0063090; right: ASGA0052511) identified in the conditional GWAS analysis.

Population	F2_LD				F2_AF				Sutai				Erhualian				Laiwu				DLY			
**Trait name**	**N**	**Mean**	**SD**	**h2**	**N**	**Mean**	**SD**	h^2	**N**	**Mean**	**SD**	h^2	**N**	**Mean**	**SD**	h^2	**N**	**Mean**	**SD**	h^2	**N**	**Mean**	**SD**	h^2
**SFA**	597	37.98	2.05	0.46	578	40.25	2.74	0.29	282	41.55	2.33	0.12	333	39.23	2.11	0.64	305	40.22	1.72	0.51	608	37.00	2.16	0.63
**MUFA**	597	48.23	3.60	0.42	578	43.76	3.81	0.35	282	47.26	4.08	0.25	333	50.68	2.19	0.64	302	51.17	1.54	0.43	555	50.68	2.11	0.49
**PUFA**	597	11.24	3.65	0.43	578	14.33	2.99	0.36	282	9.63	3.13	0.38	329	5.23	1.03	0.43	293	4.24	0.93	0.25	608	6.54	1.87	0.33
**UFA/SFA**	597	1.57	0.13	0.51	578	1.45	0.17	0.28	282	1.38	0.12	0.22	329	1.43	0.12	0.65	292	1.38	0.10	0.50	555	1.55	0.14	0.65
**PUFA/MUFA**	597	0.24	0.10	0.41	578	0.70	0.08	0.30	282	0.21	0.11	0.24	329	0.10	0.02	0.44	292	0.08	0.02	0.25	555	0.13	0.04	0.34
**MUFA/SFA**	597	1.27	0.11	0.45	578	1.10	0.15	0.30	282	1.14	0.12	0.28	333	1.30	0.12	0.67	302	1.28	0.09	0.49	555	1.37	0.12	0.65
**PUFA/SFA**	597	0.30	0.11	0.43	578	0.36	0.08	0.35	282	0.23	0.08	0.35	329	0.13	0.03	0.42	293	0.11	0.03	0.38	608	0.18	0.06	0.37
**N6**	--	--	--	--	--	---	--	--	282	9.20	2.87	0.38	330	5.07	1.00	0.43	293	4.09	0.91	0.28	561	6.32	1.84	0.32
**N3**	--	--	--	--	--	--	--	--	282	0.42	0.52	0.81	332	0.16	0.04	0.45	305	0.14	0.03	0.05	602	0.25	0.08	0.26
**N6/N3**	--	--	--	--	--	--	--	--	282	31.53	22.01	0.35	329	32.13	2.98	0.44	293	29.71	5.20	0.15	560	25.46	5.37	0.00
**C14:1n5/C14:0**	--	--	--	--	--	--	--	--	282	0.02	0.00	0.00	333	0.02	0.00	0.64	277	0.02	0.00	0.40	563	0.02	0.01	0.07
**C16:0/C14:0**	597	22.46	16.26	0.03	578	22.47	2.56	0.28	282	17.47	2.38	0.46	333	18.95	1.96	0.65	305	19.96	1.37	0.42	608	18.30	1.22	0.47
**C18:0/C16:0**	597	0.56	0.05	0.50	578	0.60	0.08	0.56	282	0.54	0.05	0.57	333	0.50	0.05	0.65	305	0.46	0.04	0.46	608	0.48	0.04	0.70
**C16:1n7/C16:0**	597	0.13	0.02	0.54	578	0.07	0.02	0.37	282	0.12	0.03	0.31	333	0.14	0.03	0.73	305	0.15	0.02	0.52	608	0.16	0.02	0.74
**C18:1n9/C16:1n7**	597	15.19	2.71	0.55	578	24.61	5.19	0.55	282	22.87	109.86	0.03	333	13.31	3.14	0.68	305	12.32	2.13	0.56	608	11.78	1.49	0.74
**C18:1n9/C18:0**	597	3.42	0.44	0.45	578	2.90	0.60	0.30	282	3.13	0.44	0.36	333	3.72	0.47	0.70	305	3.88	0.41	0.46	600	4.06	0.50	0.62
**C18:3n6/C18:2n6**	597	0.02	0.01	0.29	578	0.03	0.00	0.23	282	0.01	0.01	0.46	330	0.01	0.00	0.45	268	0.00	0.00	0.26	561	0.01	0.00	0.31
**C20:0/C18:0**	597	0.02	0.01	0.49	578	0.02	0.00	0.64	282	0.02	0.00	0.29	333	0.02	0.00	0.59	280	0.02	0.00	0.50	608	0.02	0.00	0.82
**C20:1n9/C18:1n9**	597	0.02	0.00	0.50	578	0.02	0.01	0.54	282	0.02	0.01	0.00	333	0.02	0.00	0.40	280	0.02	0.00	0.47	608	0.02	0.00	0.53
**C20:1n9/C20:0**	597	3.67	1.56	0.21	578	3.79	1.16	0.49	282	4.02	10.93	0.00	333	3.06	0.60	0.65	280	3.94	0.75	0.62	608	4.69	0.81	0.53
**C20:2n6/C18:2n6**	597	0.05	0.01	0.17	578	0.05	0.01	0.53	282	0.05	0.02	0.23	333	0.06	0.01	0.53	280	0.05	0.01	0.39	608	0.05	0.01	0.31
**C20:3n6/C18:2n6**	597	0.15	0.05	0.28	578	0.01	0.00	0.15	282	0.02	0.02	0.09	333	0.02	0.00	0.55	280	0.01	0.00	0.31	606	0.02	0.00	0.17
**C20:3n6/C18:3n6**	597	7.65	4.56	0.29	578	0.40	0.10	0.13	282	4.06	7.41	0.88	330	2.94	0.98	0.34	268	3.04	0.81	0.26	561	2.98	0.80	0.10
**C20:3n3/C18:3n3**	--	--	--	--	--	--	--	--	282	1.07	2.24	0.29	332	0.20	0.04	0.21	280	0.23	0.05	0.37	602	0.32	0.12	0.12
**C20:4n6/C18:2n6**	597	0.01	0.00	0.14	578	0.01	0.00	0.41	282	0.01	0.01	0.21	333	0.06	0.02	0.57	280	0.05	0.01	0.19	608	0.09	0.03	0.08

**C20:4n6/C18:3n6**	597	0.26	0.08	0.22	578	0.19	0.05	0.43	282	1.55	2.27	0.82	330	9.53	3.54	0.37	268	10.73	3.63	0.15	561	14.95	5.36	0.00
**C20:4n6/C20:2n6**	597	0.11	0.03	0.05	578	0.13	0.02	0.09	282	0.20	0.11	0.18	333	1.01	0.30	0.68	280	0.94	0.32	0.12	608	1.71	0.84	0.08
**C20:4n6/C20:3n6**	597	0.05	0.04	0.05	578	0.50	0.19	0.45	282	0.75	1.09	0.21	333	3.30	0.76	0.56	280	3.61	0.88	0.20	606	5.09	1.36	0.21
**FattyAI**	597	0.47	0.04	0.58	578	0.50	0.05	0.35	282	0.56	0.08	0.00	329	0.54	0.05	0.66	292	0.57	0.04	0.53	555	0.51	0.04	0.63
**FattyTI**	--	--	--	--	--	--	--	--	282	1.40	0.18	0.00	329	1.38	0.12	0.63	292	1.42	0.10	0.51	526	1.26	0.12	0.66
**ACL**	597	17.03	0.25	0.16	578	17.18	0.08	0.29	282	17.12	0.18	0.30	291	16.54	0.17	0.57	292	16.58	0.12	0.42	518	16.41	0.17	0.12
**DBI**	597	0.73	0.06	0.44	578	0.73	0.04	0.35	282	0.67	0.04	0.23	329	0.62	0.02	0.54	292	0.60	0.02	0.47	526	0.65	0.04	0.49
**UI**	597	1.46	0.11	0.44	578	1.47	0.09	0.35	282	1.35	0.08	0.23	329	1.24	0.05	0.54	292	1.21	0.04	0.47	526	1.30	0.07	0.49

Supplementary Table 1

Summary of phenotypic values in five pig populations. F_2_LD: *longissimus dorsi* samples in the White Duroc × Erhualian F_2 population; F_2_AF: abdominal fat samples in the White Duroc × Erhualian F_2 population.

Trait	Chr	SNP	Position	P-value(-log)	Weight	Direction
SFA	7	MARC0077275	17049497	4.58	2260	-----
SFA	8	MARC0029979	87866390	4.62	2261	-----
SFA	10	ALGA0058420	39211912	5.13	2260	+++++
SFA	12	MARC0085766	32562570	4.74	2256	+++-+
SFA	14	ALGA0081025	119955763	5.18	2194	-+---
SFA	14	ASGA0066073	120063463	4.63	2261	+-+++
SFA	14	ASGA0066077	120101898	6.40	2262	+++++
SFA	14	ALGA0081039	120188229	13.21	2261	+++++
SFA	14	ALGA0081040	120210617	5.85	2262	++++-
SFA	14	MARC0043866	120283339	12.76	2253	-----
SFA	14	MARC0014536	120439354	12.99	2258	+++++
SFA	14	ALGA0081091	120986865	14.17	2262	++++-
SFA	14	CASI0010164	121305916	26.78	2258	+++++
SFA	14	ALGA0081097	121330920	15.59	2262	---++
SFA	14	MARC0111695	121418492	26.46	2262	-----
SFA	14	H3GA0042070	121721589	15.62	2261	+++++
SFA	14	ASGA0066131	121782540	5.22	2262	+++-+
SFA	14	MARC0012665	121885281	15.32	2260	+++++
SFA	14	ASGA0066137	122043297	9.10	2208	++++-
SFA	14	MARC0083967	122323095	13.80	2262	++++-
SFA	14	ALGA0081147	122390223	14.90	2262	-----
SFA	14	ASGA0066158	122427131	5.20	2262	---++
SFA	14	INRA0046735	122482603	12.47	2260	++++-
SFA	14	H3GA0042098	122667984	11.44	2253	-----
SFA	14	ASGA0066162	122718232	14.67	2254	----+
SFA	14	ALGA0081164	122935134	5.84	2234	---+-
SFA	14	MARC0075113	122966625	5.61	2261	-----
SFA	14	ASGA0066177	123214577	7.04	2261	+++--
SFA	14	DIAS0004744	123268607	12.83	2260	+++++
SFA	14	MARC0031817	123290079	16.31	2259	-----
SFA	14	ALGA0081185	123378986	7.46	2261	----+
SFA	14	ASGA0066203	123757577	5.15	2262	++-++
MUFA	8	ASGA0039155	87740951	5.16	2204	+++++
MUFA	12	ALGA0120651	60466900	4.45	2203	+++++
MUFA	14	ALGA0081039	120188229	5.43	2205	-----
MUFA	14	MARC0043866	120283339	4.50	2197	+++++
MUFA	14	CASI0010164	121305916	9.52	2202	-----
MUFA	14	ALGA0081097	121330920	5.02	2206	++++-
MUFA	14	MARC0111695	121418492	9.52	2206	+++++
MUFA	14	H3GA0042070	121721589	6.87	2205	-----
MUFA	14	MARC0012665	121885281	7.78	2204	-----
MUFA	14	MARC0083967	122323095	4.62	2206	----+

MUFA	14	ALGA0081147	122390223	7.75	2206	+++++
MUFA	14	INRA0046735	122482603	6.45	2204	----+
MUFA	14	H3GA0042098	122667984	5.77	2197	++++-
MUFA	14	ASGA0066162	122718232	5.66	2198	+++++
MUFA	14	MARC0075113	122966625	4.70	2205	+++++
MUFA	14	DIAS0004744	123268607	6.36	2204	-----
MUFA	14	MARC0031817	123290079	6.65	2203	+++++
PUFA	1	ALGA0006085	152065923	4.49	2239	-----
PUFA	9	MARC0100324	129407632	4.68	2246	+++++
UFA/SFA	3	H3GA0010308	113330960	4.78	2187	+++++
UFA/SFA	8	MARC0029979	87866390	4.97	2191	+++++
UFA/SFA	10	ALGA0058420	39211912	4.49	2190	-----
UFA/SFA	14	ALGA0081025	119955763	4.90	2124	+-+++
UFA/SFA	14	ASGA0066073	120063463	4.72	2191	-+---
UFA/SFA	14	ASGA0066077	120101898	6.76	2192	-----
UFA/SFA	14	ALGA0081039	120188229	13.70	2191	-----
UFA/SFA	14	ALGA0081040	120210617	6.24	2192	----+
UFA/SFA	14	MARC0043866	120283339	12.98	2183	+++++
UFA/SFA	14	MARC0014536	120439354	12.09	2188	-----
UFA/SFA	14	ALGA0081091	120986865	12.82	2192	---++
UFA/SFA	14	CASI0010164	121305916	24.62	2188	-----
UFA/SFA	14	ALGA0081097	121330920	14.08	2192	+++-+
UFA/SFA	14	MARC0111695	121418492	24.78	2192	+++++
UFA/SFA	14	H3GA0042070	121721589	15.52	2191	-----
UFA/SFA	14	ASGA0066131	121782540	4.73	2192	--++-
UFA/SFA	14	MARC0012665	121885281	14.78	2190	-----
UFA/SFA	14	ASGA0066137	122043297	8.28	2138	----+
UFA/SFA	14	MARC0083967	122323095	12.91	2192	-----
UFA/SFA	14	ALGA0081147	122390223	14.44	2192	+++++
UFA/SFA	14	ASGA0066158	122427131	5.49	2192	+++--
UFA/SFA	14	INRA0046735	122482603	13.02	2190	-----
UFA/SFA	14	H3GA0042098	122667984	11.53	2183	+++++
UFA/SFA	14	ASGA0066162	122718232	14.85	2184	+++++
UFA/SFA	14	ALGA0081164	122935134	5.74	2164	+++-+
UFA/SFA	14	MARC0075113	122966625	6.23	2191	+++++
UFA/SFA	14	ASGA0066177	123214577	7.40	2191	---++
UFA/SFA	14	DIAS0004744	123268607	12.42	2190	-----
UFA/SFA	14	MARC0031817	123290079	15.34	2189	+++++
UFA/SFA	14	ALGA0081185	123378986	7.30	2191	+++++
UFA/SFA	14	ASGA0066203	123757577	5.25	2192	-----
PUFA/MUFA	1	ALGA0006085	152065923	4.55	2185	-----
PUFA/MUFA	9	MARC0100324	129407632	4.60	2192	+++++
MUFA/SFA	8	MARC0029979	87866390	5.21	2205	+++++

MUFA/SFA	14	ALGA0081025	119955763	6.14	2138	+++++
MUFA/SFA	14	ASGA0066073	120063463	4.76	2205	-+---
MUFA/SFA	14	ASGA0066077	120101898	7.63	2206	-----
MUFA/SFA	14	ALGA0081039	120188229	13.49	2205	-----
MUFA/SFA	14	ALGA0081040	120210617	7.81	2206	----+
MUFA/SFA	14	MARC0043866	120283339	12.19	2197	+++++
MUFA/SFA	14	MARC0014536	120439354	11.22	2202	-----
MUFA/SFA	14	ALGA0081091	120986865	10.44	2206	----+
MUFA/SFA	14	CASI0010164	121305916	22.90	2202	-----
MUFA/SFA	14	ALGA0081097	121330920	11.95	2206	+++--
MUFA/SFA	14	MARC0111695	121418492	23.13	2206	+++++
MUFA/SFA	14	H3GA0042070	121721589	13.67	2205	-----
MUFA/SFA	14	ASGA0066131	121782540	4.97	2206	---+-
MUFA/SFA	14	MARC0012665	121885281	15.15	2204	-----
MUFA/SFA	14	ASGA0066137	122043297	8.30	2152	----+
MUFA/SFA	14	MARC0083967	122323095	10.95	2206	----+
MUFA/SFA	14	ALGA0081147	122390223	14.91	2206	+++++
MUFA/SFA	14	ASGA0066158	122427131	5.90	2206	+++--
MUFA/SFA	14	INRA0046735	122482603	12.73	2204	----+
MUFA/SFA	14	H3GA0042098	122667984	11.30	2197	+++++
MUFA/SFA	14	ASGA0066162	122718232	13.18	2198	+++++
MUFA/SFA	14	ALGA0081164	122935134	6.57	2178	+++-+
MUFA/SFA	14	MARC0075113	122966625	7.40	2205	+++++
MUFA/SFA	14	ASGA0066177	123214577	6.42	2205	---++
MUFA/SFA	14	DIAS0004744	123268607	12.50	2204	-----
MUFA/SFA	14	MARC0031817	123290079	14.45	2203	+++++
MUFA/SFA	14	ALGA0081185	123378986	7.16	2205	+++++
MUFA/SFA	14	ASGA0066203	123757577	5.86	2206	-----
MUFA/SFA	14	INRA0046870	125906500	5.11	2206	+++++
PUFA/SFA	9	MARC0100324	129407632	4.69	2246	+++++
C16:0/C14:0	4	ALGA0022796	8215226	4.60	2262	-++++
C16:0/C14:0	8	ALGA0046233	7551522	13.02	2259	-----
C16:0/C14:0	8	ASGA0089198	137006114	5.48	2260	-----
C16:0/C14:0	9	ALGA0051430	12691365	6.89	2256	-----
C16:0/C14:0	9	ASGA0041665	13125438	5.31	2261	+----
C16:0/C14:0	12	H3GA0055340	79502	5.51	2262	+++++
C16:0/C14:0	12	M1GA0015685	384380	8.11	2262	+++++
C16:0/C14:0	12	MARC0091258	463662	5.94	2261	-++++
C16:0/C14:0	12	MARC0033222	724868	4.50	2256	+----
C16:0/C14:0	12	ASGA0089998	940547	7.14	2261	-----
C16:0/C14:0	12	ASGA0084331	1169606	11.92	2258	-----
C16:0/C14:0	12	ASGA0052467	1888177	4.97	2262	+++++
C16:0/C14:0	12	MARC0058650	2245718	4.53	2262	+++++

C16:0/C14:0	12	MARC0055936	3704627	5.33	2261	-----
C16:0/C14:0	12	ASGA0052546	4118938	5.43	2258	-----
C16:0/C14:0	18	ALGA0107067	38219323	6.62	2259	+++-+
C18:0/C16:0	4	ALGA0081092	63864176	7.46	2261	++++-
C18:0/C16:0	7	ALGA0039950	31500144	5.99	2262	-----
C18:0/C16:0	7	MARC0033464	35177641	5.32	2262	-----
C18:0/C16:0	8	SIRI0000509	119727822	5.41	2260	-+---
C18:0/C16:0	8	ALGA0049202	120104023	5.50	2257	+-+-+
C18:0/C16:0	8	DIAS0001763	120553940	4.66	2262	+++++
C18:0/C16:0	9	ALGA0051259	11340377	4.52	2261	+++++
C18:0/C16:0	14	MARC0034469	93590454	5.73	2255	+++++
C18:0/C16:0	14	ALGA0081025	119955763	5.02	2194	-----
C18:0/C16:0	14	ASGA0066077	120101898	7.31	2262	+++++
C18:0/C16:0	14	ALGA0081039	120188229	12.32	2261	+++++
C18:0/C16:0	14	ALGA0081040	120210617	8.05	2262	++++-
C18:0/C16:0	14	MARC0043866	120283339	12.30	2253	-----
C18:0/C16:0	14	MARC0014536	120439354	13.41	2258	+++++
C18:0/C16:0	14	ALGA0081091	120986865	16.69	2262	++++-
C18:0/C16:0	14	CASI0010164	121305916	28.94	2258	+++++
C18:0/C16:0	14	ALGA0081097	121330920	17.21	2262	---++
C18:0/C16:0	14	MARC0111695	121418492	23.84	2262	-----
C18:0/C16:0	14	H3GA0042070	121721589	20.21	2261	+++++
C18:0/C16:0	14	ASGA0066131	121782540	4.62	2262	++-++
C18:0/C16:0	14	MARC0012665	121885281	13.48	2260	+++++
C18:0/C16:0	14	ASGA0066137	122043297	13.34	2208	++++-
C18:0/C16:0	14	MARC0083967	122323095	12.98	2262	++++-
C18:0/C16:0	14	ALGA0081147	122390223	13.09	2262	-----
C18:0/C16:0	14	ASGA0066158	122427131	7.50	2262	---+-
C18:0/C16:0	14	INRA0046735	122482603	11.23	2260	++++-
C18:0/C16:0	14	H3GA0042098	122667984	9.74	2253	----+
C18:0/C16:0	14	ASGA0066162	122718232	13.78	2254	----+
C18:0/C16:0	14	ALGA0081164	122935134	5.60	2234	---+-
C18:0/C16:0	14	MARC0075113	122966625	6.99	2261	-----
C18:0/C16:0	14	ASGA0066177	123214577	8.18	2261	+++--
C18:0/C16:0	14	DIAS0004744	123268607	11.35	2260	+++++
C18:0/C16:0	14	MARC0031817	123290079	15.71	2259	-----
C16:1n7/C16:0	7	M1GA0009842	31800221	5.26	2258	-----
C16:1n7/C16:0	8	SIRI0000509	119727822	10.10	2260	+++++
C16:1n7/C16:0	8	H3GA0025322	119942184	4.62	2262	+++++
C16:1n7/C16:0	8	ALGA0049202	120104023	6.57	2257	---+-
C16:1n7/C16:0	8	DIAS0001763	120553940	5.52	2262	-----
C16:1n7/C16:0	8	ALGA0049249	120972820	5.00	2261	+++++
C16:1n7/C16:0	9	ALGA0051253	11488056	5.37	2262	+++++

C16:1n7/C16:0	9	ASGA0041538	11836128	7.24	2242	+++++
C16:1n7/C16:0	9	ASGA0041570	11950214	5.23	2262	+++++
C16:1n7/C16:0	9	ASGA0041665	13125438	5.37	2261	+++++
C16:1n7/C16:0	13	MARC0097442	34967812	4.94	2260	-----
C16:1n7/C16:0	14	ALGA0081025	119955763	4.86	2194	+++++
C16:1n7/C16:0	14	ALGA0081039	120188229	7.68	2261	-----
C16:1n7/C16:0	14	ALGA0081040	120210617	4.84	2262	----+
C16:1n7/C16:0	14	MARC0043866	120283339	7.60	2253	+++++
C16:1n7/C16:0	14	MARC0014536	120439354	6.04	2258	-----
C16:1n7/C16:0	14	ALGA0081091	120986865	8.21	2262	----+
C16:1n7/C16:0	14	CASI0010164	121305916	16.21	2258	-----
C16:1n7/C16:0	14	ALGA0081097	121330920	8.86	2262	+++--
C16:1n7/C16:0	14	MARC0111695	121418492	12.58	2262	+++-+
C16:1n7/C16:0	14	H3GA0042070	121721589	8.51	2261	-----
C16:1n7/C16:0	14	ASGA0066131	121782540	4.90	2262	-----
C16:1n7/C16:0	14	MARC0012665	121885281	9.23	2260	-----
C16:1n7/C16:0	14	ASGA0066137	122043297	6.86	2208	----+
C16:1n7/C16:0	14	MARC0083967	122323095	7.16	2262	----+
C16:1n7/C16:0	14	ALGA0081147	122390223	9.37	2262	+++++
C16:1n7/C16:0	14	ASGA0066158	122427131	4.53	2262	+++-+
C16:1n7/C16:0	14	INRA0046735	122482603	7.93	2260	----+
C16:1n7/C16:0	14	H3GA0042098	122667984	6.78	2253	++++-
C16:1n7/C16:0	14	ASGA0066162	122718232	9.03	2254	++++-
C16:1n7/C16:0	14	ALGA0081164	122935134	4.53	2234	+++-+
C16:1n7/C16:0	14	MARC0075113	122966625	5.05	2261	+++-+
C16:1n7/C16:0	14	ASGA0066177	123214577	4.75	2261	---++
C16:1n7/C16:0	14	DIAS0004744	123268607	8.19	2260	-----
C16:1n7/C16:0	14	MARC0031817	123290079	8.91	2259	+++++
C18:1n9/C16:1n7	7	ALGA0039950	31500144	5.11	2262	-----
C18:1n9/C16:1n7	7	M1GA0009842	31800221	5.38	2258	+-+++
C18:1n9/C16:1n7	8	ALGA0122904	109553065	5.50	2261	+++++
C18:1n9/C16:1n7	8	ALGA0049122	117456463	5.07	2262	++++-
C18:1n9/C16:1n7	8	ALGA0118007	119428925	4.55	2262	+++-+
C18:1n9/C16:1n7	8	SIRI0000509	119727822	18.28	2260	-----
C18:1n9/C16:1n7	8	INRA0030422	119851321	8.25	2261	+-+++
C18:1n9/C16:1n7	8	H3GA0025322	119942184	7.42	2262	-----
C18:1n9/C16:1n7	8	ALGA0049202	120104023	11.16	2257	+++-+
C18:1n9/C16:1n7	8	ASGA0091658	120254298	6.34	2262	+++++
C18:1n9/C16:1n7	8	DIAS0001763	120553940	11.36	2262	+++++
C18:1n9/C16:1n7	8	CASI0008396	120736390	5.47	2199	---+-
C18:1n9/C16:1n7	8	ALGA0049249	120972820	8.48	2261	-----
C18:1n9/C16:1n7	8	ALGA0049269	121743168	4.73	2261	+++-+
C18:1n9/C16:1n7	8	ALGA0049404	128842533	6.39	2242	--+--

C18:1n9/C16:1n7	9	ALGA0051124	10075047	5.28	2260	+++++
C18:1n9/C16:1n7	9	ALGA0051253	11488056	4.82	2262	-----
C18:1n9/C16:1n7	9	ASGA0041538	11836128	7.04	2242	-----
C18:1n9/C16:1n7	9	ASGA0041570	11950214	5.80	2262	-----
C18:1n9/C16:1n7	9	ASGA0041665	13125438	8.01	2261	-----
C18:1n9/C16:1n7	9	ASGA0041763	14350832	4.58	2262	+-+++
C18:1n9/C16:1n7	9	ALGA0051570	14512022	6.59	2262	-----
C18:1n9/C16:1n7	12	ASGA0084331	1169606	4.83	2258	-+---
C18:1n9/C16:1n7	12	ASGA0052791	7935301	4.73	2234	-+---
C18:1n9/C16:1n7	14	CASI0010164	121305916	6.55	2258	+++++
C18:1n9/C18:0	4	ALGA0081092	63864176	5.96	2253	----+
C18:1n9/C18:0	8	MARC0029979	87866390	4.63	2253	+++++
C18:1n9/C18:0	10	ALGA0058420	39211912	4.50	2252	-----
C18:1n9/C18:0	14	ASGA0091963	118732842	4.89	2253	+++--
C18:1n9/C18:0	14	CASI0007888	119514048	5.15	2253	-+-+-
C18:1n9/C18:0	14	ALGA0081025	119955763	7.25	2186	+++++
C18:1n9/C18:0	14	ASGA0066073	120063463	5.36	2253	-+---
C18:1n9/C18:0	14	ASGA0066077	120101898	9.49	2254	-----
C18:1n9/C18:0	14	ALGA0081039	120188229	16.38	2253	-----
C18:1n9/C18:0	14	ALGA0081040	120210617	9.98	2254	----+
C18:1n9/C18:0	14	MARC0043866	120283339	15.41	2245	+++++
C18:1n9/C18:0	14	MARC0014536	120439354	15.28	2250	-----
C18:1n9/C18:0	14	ALGA0081091	120986865	15.48	2254	----+
C18:1n9/C18:0	14	CASI0010164	121305916	32.15	2250	-----
C18:1n9/C18:0	14	ALGA0081097	121330920	18.25	2254	++++-
C18:1n9/C18:0	14	MARC0111695	121418492	29.42	2254	+++++
C18:1n9/C18:0	14	H3GA0042070	121721589	20.54	2253	-----
C18:1n9/C18:0	14	ASGA0066131	121782540	6.28	2254	-----
C18:1n9/C18:0	14	MARC0012665	121885281	18.51	2252	-----
C18:1n9/C18:0	14	ASGA0066137	122043297	12.74	2200	----+
C18:1n9/C18:0	14	MARC0083967	122323095	14.80	2254	----+
C18:1n9/C18:0	14	ALGA0081147	122390223	18.14	2254	+++++
C18:1n9/C18:0	14	ASGA0066158	122427131	8.00	2254	+++-+
C18:1n9/C18:0	14	INRA0046735	122482603	15.32	2252	----+
C18:1n9/C18:0	14	H3GA0042098	122667984	13.59	2245	++++-
C18:1n9/C18:0	14	ASGA0066162	122718232	16.81	2246	++++-
C18:1n9/C18:0	14	ALGA0081164	122935134	7.79	2226	+++-+
C18:1n9/C18:0	14	MARC0075113	122966625	8.80	2253	+++++
C18:1n9/C18:0	14	ASGA0066177	123214577	8.63	2253	---++
C18:1n9/C18:0	14	DIAS0004744	123268607	15.42	2252	-----
C18:1n9/C18:0	14	MARC0031817	123290079	18.96	2251	+++++
C18:1n9/C18:0	14	ALGA0081185	123378986	7.23	2253	++++-
C18:1n9/C18:0	14	DIAS0001139	123692288	4.50	2253	----+

C18:1n9/C18:0	14	ASGA0066203	123757577	5.40	2254	--+--
C18:1n9/C18:0	14	INRA0046870	125906500	4.67	2254	+++++
C20:0/C18:0	16	ASGA0072592	22691355	5.07	2221	-----
C20:0/C18:0	16	ASGA0072659	24615470	4.85	2223	+++++
C20:0/C18:0	16	H3GA0046313	25329699	6.79	2235	+++++
C20:0/C18:0	16	DRGA0015980	25960781	4.61	2236	+++++
C20:0/C18:0	16	ASGA0072743	26247215	7.38	2232	-----
C20:0/C18:0	16	ASGA0072755	26444110	4.74	2226	----+
C20:0/C18:0	16	M1GA0020968	27972337	4.67	2237	----+
C20:0/C18:0	16	ASGA0096846	28409425	5.82	2237	--+--
C20:0/C18:0	16	ALGA0107886	28627959	4.58	2237	+++++
C20:0/C18:0	16	DIAS0002738	29568619	5.48	2230	-----
C20:0/C18:0	16	H3GA0046366	29952113	5.51	2237	+++++
C20:0/C18:0	16	MARC0013435	30289787	4.57	2215	--+--
C20:0/C18:0	16	DRGA0016027	30515922	9.08	2235	+++++
C20:0/C18:0	16	MARC0032144	30669371	7.30	2237	++++-
C20:0/C18:0	16	H3GA0046376	30709921	5.11	2232	+++++
C20:0/C18:0	16	ASGA0084351	30956011	9.14	2236	++-++
C20:0/C18:0	16	MARC0112978	30967634	5.54	2236	--+--
C20:0/C18:0	16	ALGA0090009	31016837	7.38	2235	-++--
C20:0/C18:0	16	ALGA0090017	31103652	5.15	2237	----+
C20:0/C18:0	16	ALGA0090018	31129915	10.94	2235	-----
C20:0/C18:0	16	ALGA0090020	31213359	6.17	2232	++++-
C20:0/C18:0	16	ALGA0107417	31684786	5.21	2235	+----
C20:0/C18:0	16	ALGA0105400	31856522	5.32	2237	++++-
C20:0/C18:0	16	DRGA0016047	31961254	5.85	2237	----+
C20:0/C18:0	16	ALGA0090076	32979912	7.65	2237	-----
C20:0/C18:0	16	MARC0020510	33672880	13.48	2236	+++++
C20:0/C18:0	16	ALGA0090112	33905749	11.37	2236	-----
C20:0/C18:0	16	ALGA0090119	34003949	6.00	2237	++++-
C20:0/C18:0	16	ALGA0090125	34056662	9.81	2236	++-++
C20:0/C18:0	16	ALGA0090122	34122397	10.27	2237	+++++
C20:0/C18:0	16	CASI0008095	34194345	10.17	2236	+++++
C20:0/C18:0	16	ASGA0072949	34715842	5.58	2237	-+---
C20:0/C18:0	16	ASGA0095837	35014537	5.33	2235	-----
C20:0/C18:0	16	ASGA0099845	35035130	5.33	2237	+++++
C20:0/C18:0	16	ASGA0072968	35190026	15.70	2225	--+--
C20:0/C18:0	16	ALGA0090171	35212148	10.23	2234	++-++
C20:0/C18:0	16	DRGA0016089	35289193	14.38	2236	--+--
C20:0/C18:0	16	ALGA0090177	35333101	8.21	2236	++-++
C20:0/C18:0	16	ASGA0073002	35661077	13.16	2227	+++++
C20:0/C18:0	16	ALGA0090229	35839844	5.41	2237	++-++
C20:0/C18:0	16	H3GA0046461	36289858	4.81	2236	--+--

C20:0/C18:0	16	ALGA0090273	36472679	7.25	2221	+++++
C20:0/C18:0	16	ALGA0090266	36506690	11.62	2236	-----
C20:0/C18:0	16	ALGA0116942	36852067	6.08	2184	+-+-+
C20:0/C18:0	16	MARC0027943	36933143	4.62	2237	---+-
C20:0/C18:0	16	ASGA0073070	37408125	4.79	2222	+-+-+
C20:0/C18:0	16	M1GA0020993	37423396	8.65	2233	--++-
C20:0/C18:0	16	ASGA0073065	37584102	4.71	2237	+++--
C20:0/C18:0	16	ALGA0112710	37673844	8.89	2237	-+---
C20:0/C18:0	16	M1GA0020996	38260722	10.59	2236	+-+++
C20:0/C18:0	16	DRGA0016119	38367050	5.78	2213	+-+++
C20:0/C18:0	16	ASGA0097154	38383883	4.64	2237	+-+-+
C20:0/C18:0	16	MARC0013760	38472566	5.23	2224	+++-+
C20:0/C18:0	16	ALGA0090355	38745958	12.71	2236	+++-+
C20:0/C18:0	16	DRGA0016122	38814746	9.04	2237	++--+
C20:0/C18:0	16	ALGA0090370	38964196	7.64	2237	+++-+
C20:0/C18:0	16	ALGA0090377	39171047	10.76	2229	-----
C20:0/C18:0	16	ALGA0090392	40150018	11.04	2237	+++-+
C20:0/C18:0	16	ALGA0090412	40783785	4.46	2237	-+---
C20:0/C18:0	16	MARC0084747	40932270	33.94	2237	+++++
C20:0/C18:0	16	ALGA0090426	41305472	19.94	2237	----+
C20:0/C18:0	16	ALGA0090423	41393886	13.11	2236	--++-
C20:0/C18:0	16	ALGA0090435	41627410	15.14	2233	+++++
C20:0/C18:0	16	ALGA0090469	42269129	20.52	2237	+++++
C20:0/C18:0	16	ALGA0090487	43093614	12.69	2237	+++--
C20:0/C18:0	16	ASGA0073192	43402965	13.79	2237	---++
C20:0/C18:0	16	DRGA0016154	43516213	28.52	2215	+++++
C20:0/C18:0	16	DRGA0016155	43534471	51.47	2237	+++++
C20:0/C18:0	16	MARC0051739	44429692	32.62	2237	---+-
C20:0/C18:0	16	ASGA0073222	44919236	16.17	2237	+++--
C20:0/C18:0	16	DRGA0016166	45154456	22.77	2236	+++++
C20:0/C18:0	16	DRGA0016169	45313348	19.81	2236	++++-
C20:0/C18:0	16	ALGA0090519	45576077	7.93	2237	++++-
C20:0/C18:0	16	DRGA0016176	46066010	22.75	2237	++++-
C20:0/C18:0	16	ASGA0097650	46602679	6.09	2237	-----
C20:0/C18:0	16	ASGA0073240	46868015	6.36	2215	+++-+
C20:0/C18:0	16	ASGA0073242	46893077	16.44	2236	-----
C20:0/C18:0	16	SIRI0000384	47011906	20.53	2237	-----
C20:0/C18:0	16	MARC0026216	47630018	8.48	2236	++-++
C20:0/C18:0	16	ASGA0073252	47650300	8.17	2210	++-++
C20:0/C18:0	16	ASGA0073269	48069505	4.69	2236	-+-+-
C20:0/C18:0	16	ASGA0073261	48130360	10.16	2232	++-++
C20:0/C18:0	16	ASGA0073272	48203983	43.13	2235	+++++
C20:0/C18:0	16	MARC0093848	48917468	5.15	2236	----+

C20:0/C18:0	16	DRGA0016198	48972181	6.11	2237	---+-
C20:0/C18:0	16	ALGA0090620	49083460	9.72	2236	++++-
C20:0/C18:0	16	MARC0098527	49178924	7.46	2237	+++-+
C20:0/C18:0	16	ASGA0084765	49185777	15.32	2237	+++++
C20:0/C18:0	16	ALGA0090646	50417558	6.03	2237	++++-
C20:0/C18:0	16	ALGA0090652	50786940	13.84	2236	----+
C20:0/C18:0	16	ALGA0090674	51865432	12.41	2236	+++-+
C20:0/C18:0	16	ISU10000643	51990539	7.16	2237	-----
C20:0/C18:0	16	M1GA0021013	52012266	7.41	2235	+++++
C20:0/C18:0	16	H3GA0046599	52206927	5.76	2236	+++-+
C20:0/C18:0	16	ASGA0073361	52416884	6.09	2226	---+-
C20:0/C18:0	16	MARC0049832	52439349	6.86	2236	--+--
C20:0/C18:0	16	ASGA0090510	53481413	5.76	2234	++-++
C20:0/C18:0	16	ALGA0121984	53501437	5.72	2214	++-++
C20:0/C18:0	16	ASGA0086954	53505277	4.57	2228	++-++
C20:0/C18:0	16	H3GA0046623	54031268	4.80	2237	+++-+
C20:0/C18:0	16	ALGA0090725	54325792	6.98	2237	+++++
C20:0/C18:0	16	ASGA0073382	55361884	6.42	2237	++-++
C20:0/C18:0	16	MARC0005681	55691446	9.06	2231	--+--
C20:0/C18:0	16	MARC0008457	55767594	4.58	2233	-----
C20:0/C18:0	16	ASGA0073404	55993053	6.37	2237	--+--
C20:0/C18:0	16	ALGA0090772	56916532	4.96	2237	+++++
C20:0/C18:0	16	H3GA0046657	56936736	6.44	2237	-----
C20:0/C18:0	16	H3GA0046765	59605947	5.19	2187	++++-
C20:0/C18:0	16	ASGA0073613	60183758	4.63	2230	+++++
C20:0/C18:0	16	ALGA0108312	60401023	4.88	2237	-----
C20:0/C18:0	16	ASGA0073651	61995301	4.61	2237	++++-
C20:0/C18:0	16	DRGA0016251	63229295	4.66	2231	-----
C20:0/C18:0	16	MARC0010374	67857563	5.33	2218	+++++
C20:0/C18:0	16	ASGA0073800	69095599	5.83	2236	-----
C20:0/C18:0	16	ASGA0073815	69294616	5.36	2236	+++++
C20:0/C18:0	16	ASGA0073817	69305036	4.71	2216	+++++
C20:0/C18:0	16	MARC0109000	69503193	5.66	2237	+++++
C20:1n9/C18:1n9	7	ASGA0032266	32543114	8.10	2220	-----
C20:1n9/C18:1n9	7	ASGA0032270	32786027	6.56	2234	++-++
C20:1n9/C18:1n9	7	ASGA0032276	32808311	8.19	2237	+++++
C20:1n9/C18:1n9	7	MARC0074480	42999450	4.62	2236	----+
C20:1n9/C18:1n9	7	ALGA0040856	43166101	4.57	2230	++++-
C20:1n9/C18:1n9	7	DRGA0007537	48920722	4.92	2235	-----
C20:1n9/C18:1n9	7	ALGA0041169	49071381	7.74	2230	-----
C20:1n9/C18:1n9	7	MARC0083963	50138934	4.76	2237	+++-+
C20:1n9/C18:1n9	7	ALGA0041338	50844200	6.38	2233	--+--
C20:1n9/C18:1n9	7	ALGA0041419	51894605	5.13	2237	++-++

C20:1n9/C18:1n9	7	ALGA0041464	52279313	8.69	2234	----+
C20:1n9/C18:1n9	7	MARC0034834	53102034	8.06	2237	----+
C20:1n9/C18:1n9	7	ALGA0041580	53559770	6.73	2237	+++++
C20:1n9/C18:1n9	7	ALGA0041598	53707250	9.56	2235	-----
C20:1n9/C18:1n9	7	H3GA0055560	54997566	6.13	2180	+++-+
C20:1n9/C18:1n9	7	M1GA0010388	55681553	4.63	2234	+++++
C20:1n9/C18:1n9	7	ALGA0041948	55878376	5.20	2235	-++--
C20:1n9/C18:1n9	7	ALGA0041977	56166104	8.13	2212	+-+++
C20:1n9/C18:1n9	7	ASGA0034040	56199605	6.80	2237	+-+++
C20:1n9/C18:1n9	7	ASGA0034043	56215710	6.63	2237	---+-
C20:1n9/C18:1n9	7	ASGA0034046	56230077	4.51	2236	-----
C20:1n9/C18:1n9	7	INRA0025982	56849029	5.06	2235	+++++
C20:1n9/C18:1n9	7	H3GA0021788	56888377	5.20	2237	-+---
C20:1n9/C18:1n9	7	ASGA0034092	56916492	5.84	2236	-----
C20:1n9/C18:1n9	7	ALGA0042183	59039109	4.55	2236	+++++
C20:1n9/C18:1n9	7	ASGA0034251	62438211	5.07	2237	-----
C20:1n9/C18:1n9	7	MARC0067183	70357011	4.68	2230	+-+++
C20:1n9/C18:1n9	7	ALGA0042424	70401988	4.80	2237	+++++
C20:1n9/C18:1n9	7	ALGA0042516	74587760	4.72	2236	++-++
C20:1n9/C18:1n9	7	ALGA0042635	80902468	4.86	2236	----+
C20:1n9/C18:1n9	7	ALGA0043757	99048843	5.00	2235	-----
C20:1n9/C18:1n9	7	ALGA0114746	134540651	8.97	2234	+--++
C20:1n9/C18:1n9	16	MARC0020510	33672880	5.08	2236	+++++
C20:1n9/C18:1n9	16	MARC0084747	40932270	6.58	2237	+++++
C20:1n9/C18:1n9	16	DRGA0016155	43534471	4.56	2237	+++++
C20:1n9/C20:0	7	ASGA0032266	32543114	4.53	2220	-----
C20:1n9/C20:0	7	ASGA0032276	32808311	6.32	2237	+++++
C20:1n9/C20:0	7	MARC0083963	50138934	6.37	2237	+-+++
C20:1n9/C20:0	7	ALGA0041338	50844200	4.60	2233	-----
C20:1n9/C20:0	7	ALGA0041464	52279313	4.78	2234	----+
C20:1n9/C20:0	7	ASGA0033595	52462626	5.03	2187	+++++
C20:1n9/C20:0	7	ALGA0041948	55878376	6.41	2235	--+--
C20:1n9/C20:0	7	H3GA0021739	55905779	5.76	2232	++-++
C20:1n9/C20:0	7	ASGA0034040	56199605	4.45	2237	+-+++
C20:1n9/C20:0	7	ALGA0114746	134540651	7.12	2234	+--++
C20:1n9/C20:0	14	ASGA0061202	11325047	5.53	2237	---+-
C20:1n9/C20:0	14	CASI0010164	121305916	5.50	2233	-----
C20:1n9/C20:0	14	MARC0111695	121418492	6.60	2237	+++++
C20:1n9/C20:0	16	ASGA0103355	20271819	4.46	2236	+++++
C20:1n9/C20:0	16	ASGA0072592	22691355	4.91	2221	+-+++
C20:1n9/C20:0	16	ALGA0090009	31016837	4.60	2235	+--++
C20:1n9/C20:0	16	ALGA0090112	33905749	4.88	2236	+++++
C20:1n9/C20:0	16	ASGA0073002	35661077	4.55	2227	---+-

C20:1n9/C20:0	16	ALGA0090273	36472679	5.46	2221	---+-
C20:1n9/C20:0	16	ALGA0112710	37673844	4.89	2237	+--++
C20:1n9/C20:0	16	ALGA0090355	38745958	7.00	2236	---+-
C20:1n9/C20:0	16	DRGA0016122	38814746	5.58	2237	---+-
C20:1n9/C20:0	16	MARC0084747	40932270	10.85	2237	-----
C20:1n9/C20:0	16	ALGA0090426	41305472	4.94	2237	+++--
C20:1n9/C20:0	16	ALGA0090423	41393886	4.46	2236	++--+
C20:1n9/C20:0	16	ALGA0090435	41627410	4.61	2233	----+
C20:1n9/C20:0	16	ALGA0090469	42269129	5.35	2237	---++
C20:1n9/C20:0	16	ASGA0073192	43402965	6.61	2237	+++--
C20:1n9/C20:0	16	DRGA0016154	43516213	8.94	2215	---+-
C20:1n9/C20:0	16	DRGA0016155	43534471	17.01	2237	-----
C20:1n9/C20:0	16	MARC0051739	44429692	13.05	2237	+++-+
C20:1n9/C20:0	16	ASGA0073222	44919236	7.76	2237	---++
C20:1n9/C20:0	16	DRGA0016166	45154456	9.21	2236	---+-
C20:1n9/C20:0	16	DRGA0016169	45313348	7.41	2236	----+
C20:1n9/C20:0	16	DRGA0016176	46066010	6.96	2237	----+
C20:1n9/C20:0	16	ASGA0097650	46602679	4.64	2237	+++++
C20:1n9/C20:0	16	ASGA0073242	46893077	7.22	2236	+++-+
C20:1n9/C20:0	16	SIRI0000384	47011906	7.90	2237	+++-+
C20:1n9/C20:0	16	MARC0026216	47630018	5.24	2236	-----
C20:1n9/C20:0	16	ASGA0073252	47650300	5.01	2210	-----
C20:1n9/C20:0	16	ASGA0073272	48203983	17.10	2235	-----
C20:1n9/C20:0	16	DRGA0016198	48972181	5.69	2237	+++--
C20:1n9/C20:0	16	ALGA0090620	49083460	6.36	2236	----+
C20:1n9/C20:0	16	MARC0098527	49178924	5.25	2237	---+-
C20:1n9/C20:0	16	ASGA0084765	49185777	8.11	2237	-+---
C20:1n9/C20:0	16	ALGA0090674	51865432	5.19	2236	---+-
C20:1n9/C20:0	16	ASGA0073361	52416884	6.44	2226	+++-+
C20:1n9/C20:0	16	H3GA0046668	57488206	4.93	2237	+++++
C20:1n9/C20:0	16	ALGA0090834	58445833	5.04	2235	-----
C20:1n9/C20:0	16	H3GA0046765	59605947	5.42	2187	----+
C20:1n9/C20:0	16	ASGA0105593	61213135	4.91	2236	-----
C20:4n6/C20:2n6	1	MARC0044150	286933	4.59	2237	-----
C20:4n6/C20:2n6	4	ALGA0026828	101428842	4.69	2236	-----
C20:4n6/C20:2n6	5	H3GA0016834	75797340	5.16	2236	-----
C20:4n6/C20:2n6	7	MARC0037137	23206164	4.56	2236	+-+++
C20:4n6/C20:2n6	7	MARC0033464	35177641	4.79	2237	+++++
FattyAI	4	MARC0029984	112290496	4.63	2191	+++++
FattyAI	8	DIAS0001763	120553940	5.52	2192	-+---
FattyAI	8	ASGA0092052	123806415	4.61	2181	+++++
FattyAI	10	MARC0097643	40186438	4.76	2191	-----
FattyAI	14	ALGA0081039	120188229	4.74	2191	+++++

FattyAI	14	ALGA0081091	120986865	5.11	2192	+++--
FattyAI	14	CASI0010164	121305916	8.69	2188	+++++
FattyAI	14	ALGA0081097	121330920	5.82	2192	-----
FattyAI	14	MARC0111695	121418492	9.77	2192	-----
FattyAI	14	H3GA0042070	121721589	5.34	2191	+++++
FattyAI	14	MARC0012665	121885281	5.50	2190	+++++
FattyAI	14	MARC0083967	122323095	5.17	2192	+++++
FattyAI	14	ALGA0081147	122390223	5.35	2192	-----
FattyAI	14	INRA0046735	122482603	5.16	2190	+++++
FattyAI	14	H3GA0042098	122667984	4.62	2183	-----
FattyAI	14	ASGA0066162	122718232	5.84	2184	-----
FattyAI	14	DIAS0004744	123268607	4.66	2190	+++++
FattyAI	14	MARC0031817	123290079	5.93	2189	-----
FattyAI	18	ASGA0078884	11891293	4.89	2167	-----
ACL	4	H3GA0014062	118534475	6.06	2116	+++++
ACL	8	ASGA0039671	121558064	5.89	2083	-+---
DBI	14	ALGA0081091	120986865	5.21	2163	---+-
DBI	14	CASI0010164	121305916	6.99	2159	-----
DBI	14	ALGA0081097	121330920	5.01	2163	+++-+
DBI	14	MARC0111695	121418492	7.07	2163	+++++
DBI	14	H3GA0042070	121721589	6.09	2162	+----
DBI	14	MARC0083967	122323095	5.49	2163	-----
DBI	14	ASGA0066162	122718232	5.28	2155	+++++
DBI	14	MARC0031817	123290079	4.97	2160	+++++
UI	14	ALGA0081091	120986865	5.21	2163	---+-
UI	14	CASI0010164	121305916	6.99	2159	-----
UI	14	ALGA0081097	121330920	5.01	2163	+++-+
UI	14	MARC0111695	121418492	7.07	2163	+++++
UI	14	H3GA0042070	121721589	6.09	2162	+----
UI	14	MARC0083967	122323095	5.49	2163	-----
UI	14	ASGA0066162	122718232	5.28	2155	+++++
UI	14	MARC0031817	123290079	4.97	2160	+++++
N6	9	MARC0100324	129407632	4.60	1603	++++
N3	9	MARC0100324	129407632	5.17	1658	++++
N3	15	ALGA0123434	1443718	5.25	1658	----
N3	15	MARC0034611	4129181	4.65	1658	----
N6/N3	15	ALGA0123434	1443718	5.45	1601	++++
C14:1n5/C14:0	7	M1GA0009842	31800221	5.53	1588	----
C14:1n5/C14:0	7	MARC0069646	34103417	4.66	1592	----
C14:1n5/C14:0	12	ALGA0122316	3663932	4.61	1568	++++
C14:1n5/C14:0	12	ALGA0123789	13486734	4.48	1592	----
C14:1n5/C14:0	14	CASI0010164	121305916	6.29	1588	----
C18:3n6/C18:2n6	7	DRGA0007253	18714004	6.98	1577	++++

C18:3n6/C18:2n6	7	ASGA0031579	18822579	6.84	1576	----
C18:3n6/C18:2n6	11	ASGA0095757	25924767	4.58	1578	++++
C18:3n6/C18:2n6	17	INRA0053121	26071970	4.72	1570	----
C20:2n6/C18:2n6	7	INRA0024482	29619407	5.03	1639	++++
C20:2n6/C18:2n6	7	ALGA0040094	33574487	5.89	1638	++++
C20:2n6/C18:2n6	7	MARC0002603	43780108	4.63	1639	----
C20:2n6/C18:2n6	7	MARC0034834	53102034	4.86	1640	---+
C20:2n6/C18:2n6	7	ALGA0041580	53559770	5.11	1640	++++
C20:2n6/C18:2n6	7	ALGA0041977	56166104	4.50	1616	-+++
C20:2n6/C18:2n6	7	MARC0039911	134146665	5.51	1640	+---
C20:3n6/C18:2n6	2	ASGA0008883	9260718	10.16	1638	++++
C20:3n6/C18:2n6	2	MARC0067088	12458499	5.72	1635	++++
C20:3n6/C18:2n6	9	INRA0032055	82621184	5.35	1637	++++
C20:3n6/C18:2n6	9	MARC0063505	117668749	4.77	1638	----
C20:3n6/C18:3n6	7	DRGA0007253	18714004	4.95	1577	----
C20:3n6/C18:3n6	7	ASGA0031579	18822579	6.49	1576	++++
C20:3n6/C18:3n6	7	M1GA0027226	30592663	5.94	1573	----
C20:3n6/C18:3n6	7	INRA0024930	39211095	4.75	1575	----
C20:3n6/C18:3n6	7	ASGA0034040	56199605	4.81	1578	++++
C20:3n6/C18:3n6	7	ALGA0043842	100195980	4.71	1551	++++
C20:3n3/C18:3n3	7	ASGA0032276	32808311	6.18	1633	-+++
C20:3n3/C18:3n3	7	H3GA0021439	51991520	6.55	1629	-+++
C20:3n3/C18:3n3	7	H3GA0021787	56866248	5.20	1633	++++
C20:4n6/C18:3n6	1	ASGA0004231	121663779	5.09	1573	----
C20:4n6/C18:3n6	17	MARC0061919	16920977	4.68	1570	----
C20:4n6/C20:3n6	1	MARC0044150	286933	5.57	1638	----
C20:4n6/C20:3n6	2	ASGA0008884	9139348	5.98	1634	----
C20:4n6/C20:3n6	2	MARC0067088	12458499	6.01	1635	----
C20:4n6/C20:3n6	2	ALGA0012169	17537218	4.54	1637	----
C20:4n6/C20:3n6	4	MARC0030157	98562527	6.14	1637	++++
C20:4n6/C20:3n6	4	ASGA0020851	98609935	6.24	1638	++++
C20:4n6/C20:3n6	12	ALGA0114393	58472986	4.79	1627	----
C20:4n6/C20:3n6	13	DRGA0012815	122198084	5.04	1635	----
FattyTI	3	H3GA0010308	113330960	5.19	1562	----
FattyTI	4	MARC0052574	63982652	4.67	1566	++-+
FattyTI	14	ALGA0081039	120188229	8.20	1565	++++
FattyTI	14	ALGA0081040	120210617	4.56	1566	+++-
FattyTI	14	MARC0043866	120283339	7.54	1565	----
FattyTI	14	MARC0014536	120439354	7.44	1562	++++
FattyTI	14	ALGA0081091	120986865	10.52	1566	++--
FattyTI	14	CASI0010164	121305916	17.17	1562	++++
FattyTI	14	ALGA0081097	121330920	11.39	1566	--+-
FattyTI	14	MARC0111695	121418492	17.28	1566	----

FattyTI	14	ASGA0066116	121467984	10.98	1566	---+
FattyTI	14	ALGA0081112	121527590	8.33	1566	++++
FattyTI	14	H3GA0042070	121721589	13.91	1565	++++
FattyTI	14	MARC0012665	121885281	8.60	1564	++++
FattyTI	14	ASGA0066137	122043297	6.81	1524	+++-
FattyTI	14	MARC0083967	122323095	9.29	1566	++++
FattyTI	14	ALGA0081147	122390223	8.20	1566	----
FattyTI	14	INRA0046735	122482603	6.96	1564	++++
FattyTI	14	H3GA0042098	122667984	6.00	1563	----
FattyTI	14	ASGA0066162	122718232	8.88	1563	----
FattyTI	14	MARC0091464	122956381	7.17	1565	++++
FattyTI	14	ASGA0066177	123214577	5.11	1565	++--
FattyTI	14	DIAS0004744	123268607	6.86	1564	++++
FattyTI	14	MARC0031817	123290079	11.57	1563	----

Supplementary Table 2

Significant loci surpassing the suggestive threshold in the meta-analysis across five populations. Chr: chromosome; Position: the genomic position on the *Sscrofa* 10.2 pig genome assembly; *P*-value: the statistical *P* values based on the sample size weighted analysis; Weight: the number of individuals contributing to the particular *P*-value; Direction: the corresponding direction of the effect for the QTL allele on F_2, Sutai, DLY, Erhualian and Laiwu pigs, respectively; +, positive effect; -, negative effect.

Feature	FDR	Genes in network	Genes in genome
fatty acid metabolic process	1.17E-18	18	209
acylglycerol metabolic process	3.16E-16	13	85
neutral lipid metabolic process	3.16E-16	13	85
triglyceride metabolic process	6.68E-15	12	79
long-chain fatty-acyl-CoA metabolic process	6.68E-15	9	22
fatty-acyl-CoA metabolic process	1.46E-14	9	24
fatty-acyl-CoA biosynthetic process	9.02E-14	8	17
long-chain fatty-acyl-CoA biosynthetic process	9.02E-14	8	17
triglyceride biosynthetic process	1.62E-13	10	50
neutral lipid biosynthetic process	2.03E-13	10	52
acylglycerol biosynthetic process	2.03E-13	10	52
fatty acid biosynthetic process	2.20E-13	11	79
thioester biosynthetic process	1.63E-11	8	31
acyl-CoA biosynthetic process	1.63E-11	8	31
acyl-CoA metabolic process	2.09E-11	9	54
thioester metabolic process	2.09E-11	9	54
monocarboxylic acid biosynthetic process	2.66E-11	11	124
very long-chain fatty acid metabolic process	8.15E-11	7	21
glycerolipid metabolic process	2.42E-10	13	271
carboxylic acid biosynthetic process	1.71E-09	11	184
organic acid biosynthetic process	1.71E-09	11	184
coenzyme metabolic process	1.84E-09	10	133
coenzyme biosynthetic process	3.48E-09	8	61
cholesterol metabolic process	9.31E-09	8	69
glycerolipid biosynthetic process	9.58E-09	11	219
long-chain fatty acid metabolic process	1.71E-08	8	75
cofactor metabolic process	2.62E-08	10	177
sterol metabolic process	2.70E-08	8	80
unsaturated fatty acid metabolic process	2.89E-08	8	81
cofactor biosynthetic process	3.41E-08	8	83
cholesterol biosynthetic process	1.84E-07	6	33
sterol biosynthetic process	2.16E-07	6	34
alpha-linolenic acid metabolic process	8.63E-06	4	11
linoleic acid metabolic process	8.63E-06	4	11
transferase activity, transferring acyl groups other than amino-acyl groups	1.85E-05	7	121
steroid metabolic process	2.68E-05	8	196
transferase activity, transferring acyl groups	5.82E-05	7	144
alcohol biosynthetic process	1.50E-04	6	101
steroid biosynthetic process	2.05E-04	6	107
alcohol metabolic process	2.36E-04	8	264
organic hydroxy compound biosynthetic process	4.89E-04	6	125

lipid particle	1.83E-03	4	40
lipid storage	2.39E-03	4	43
neutral lipid catabolic process	1.54E-02	3	24
acylglycerol catabolic process	1.54E-02	3	24
regulation of lipid biosynthetic process	2.54E-02	4	79
regulation of lipid metabolic process	3.03E-02	5	162
neuronal action potential	3.15E-02	3	31
glycerolipid catabolic process	3.40E-02	3	32
regulation of lipid storage	4.01E-02	3	34
integral component of endoplasmic reticulum membrane	4.51E-02	4	94
intrinsic component of endoplasmic reticulum membrane	4.60E-02	4	95
axon ensheathment	4.79E-02	3	37
ensheathment of neurons	4.79E-02	3	37
unsaturated fatty acid biosynthetic process	7.39E-02	3	43
cellular lipid catabolic process	8.34E-02	4	113

Supplementary Table 3.

Functional enrichment analysis involved by the candidate genes in this study. Feature is the biological pathway name involved by the candidate genes; FDR: false discovery rate.

Group	Name	Weight	PubMed	Source
Co-expression	Innocenti-Brown-2011	11.97%	http://www.ncbi.nlm.nih.gov/pubmed/21637794	Pearson correlation with 579,361 interactions from GEO
Co-expression	Mallon-McKay-2013	6.65%	http://www.ncbi.nlm.nih.gov/pubmed/23117585	Pearson correlation with 567,140 interactions from GEO
Co-expression	Smirnov-Cheung-2009	6.15%	http://www.ncbi.nlm.nih.gov/pubmed/19349959	Pearson correlation with 463,390 interactions from GEO
Co-expression	Chen-Brown-2002	6.05%	http://www.ncbi.nlm.nih.gov/pubmed/12058060	Pearson correlation with 275,649 interactions from supplementary material
Co-expression	Roth-Zlotnik-2006	5.92%	http://www.ncbi.nlm.nih.gov/pubmed/16572319	Pearson correlation with 666,614 interactions from GEO
Co-expression	Rieger-Chu-2004	5.61%	http://www.ncbi.nlm.nih.gov/pubmed/15096622	Pearson correlation with 259,055 interactions from GEO
Co-expression	Burington-Shaughnessy-2008	3.72%	http://www.ncbi.nlm.nih.gov/pubmed/18676754	Pearson correlation with 293,587 interactions from GEO
Co-expression	Gysin-McMahon-2012	2.21%	http://www.ncbi.nlm.nih.gov/pubmed/22833572	Pearson correlation with 388,454 interactions from GEO
Co-expression	Salaverria-Siebert-2011	2.11%	http://www.ncbi.nlm.nih.gov/pubmed/21487109	Pearson correlation with 514,070 interactions from GEO
Co-expression	Arijs-Rutgeerts-2009	1.16%	http://www.ncbi.nlm.nih.gov/pubmed/19956723	Pearson correlation with 653,194 interactions from GEO
Co-expression	Bild-Nevins-2006 B	1.00%	http://www.ncbi.nlm.nih.gov/pubmed/16273092	Pearson correlation with 282,582 interactions from GEO
Co-expression	Cheok-Evans-2003	0.68%	http://www.ncbi.nlm.nih.gov/pubmed/12704389	Pearson correlation with 263,940 interactions from GEO
Co-expression	Kang-Willman-2010	0.55%	http://www.ncbi.nlm.nih.gov/pubmed/19880498	Pearson correlation with 656,632 interactions from GEO
Co-expression	Boldrick-Relman-2002	0.54%	http://www.ncbi.nlm.nih.gov/pubmed/11805339	Pearson correlation with 108,543 interactions from supplementary material
Co-expression	Bahr-Bowler-2013	0.30%	http://www.ncbi.nlm.nih.gov/pubmed/23590301	Pearson correlation with 278,447 interactions from GEO
Co-expression	Perou-Botstein-1999	0.24%	http://www.ncbi.nlm.nih.gov/pubmed/10430922	Pearson correlation with 62,886 interactions from supplementary material
Co-expression	Wu-Garvey-2007	0.04%	http://www.ncbi.nlm.nih.gov/pubmed/17709892	Pearson correlation with 260,762 interactions from GEO

Co-expression	Wang-Maris-2006	0.02%	http://www.ncbi.nlm.nih.gov/pubmed/16778177	Pearson correlation with 264,234 interactions from GEO
Co-localization	Johnson-Shoemaker-2003	3.86%	http://www.ncbi.nlm.nih.gov/pubmed/14684825	Pearson correlation with 426,640 interactions from GEO
Genetic interactions	Lin-Smith-2010	0.23%	http://www.ncbi.nlm.nih.gov/pubmed/20508145	with 4,836,794 interactions from supplementary material
Pathway	Wu-Stein-2010	7.11%	http://www.ncbi.nlm.nih.gov/pubmed/20482850	with 78,183 interactions from supplementary material
Physical interactions	Albers-Koegl-2005	0.34%	http://www.ncbi.nlm.nih.gov/pubmed/15604093	Direct interaction with 327 interactions from iRefIndex
Physical interactions	IREF-BIND	0.04%		Direct interaction with 7,078 interactions from iRefIndex
Predicted	I2D-vonMering-Bork-2002-Medium-Yeast2Human	15.70%	http://www.ncbi.nlm.nih.gov/pubmed/12000970	Direct interaction with 3,001 interactions from I2D
Predicted	I2D-BioGRID-Yeast2Human	5.87%	http://www.ncbi.nlm.nih.gov/pubmed/16381927	Direct interaction with 13,302 interactions from I2D
Shared protein domains	INTERPRO	8.19%		Pearson correlation with 562,560 interactions from InterPro
Shared protein domains	PFAM	3.73%		Pearson correlation with 543,464 interactions from Pfam

Supplementary Table 4.

Network enrichment analysis for candidate genes highlighted in this study.
